# Identification and Classification of Differentially Expressed Genes and Network Meta-Analysis Reveals Potential Molecular Signatures Associated With Tuberculosis

**DOI:** 10.3389/fgene.2019.00932

**Published:** 2019-11-04

**Authors:** Aftab Alam, Nikhat Imam, Mohd Murshad Ahmed, Safia Tazyeen, Naaila Tamkeen, Anam Farooqui, Md. Zubbair Malik, Romana Ishrat

**Affiliations:** ^1^Centre for Interdisciplinary Research in Basic Sciences, Jamia Millia Islamia, New Delhi, India; ^2^Department of Mathematics, Institute of Computer Science & Information Technology, Magadh University, Bodh Gaya, India; ^3^Department of Biosciences, Jamia Millia Islamia, New Delhi, India; ^4^School of Computational & Integrative Sciences, Jawaharlal Nehru University, New Delhi, India

**Keywords:** meta-analysis, DEGs, gene ontology, KEGG, network analysis, gene knock-out, LCP

## Abstract

Tuberculosis (TB) is one of deadly transmissible disease that causes death worldwide; however, only 10% of people infected with *Mycobacterium* *tuberculosis* develop disease, indicating that host genetic factors may play key role in determining susceptibility to TB disease. In this way, the analysis of gene expression profiling of TB infected individuals can give us a snapshot of actively expressed genes and transcripts under various conditions. In the present study, we have analyzed microarray data set and compared the gene expression profiles of patients with different datasets of healthy control, latent infection, and active TB. We observed the transition of genes from normal condition to different stages of the TB and identified and annotated those genes/pathways/processes that have important roles in TB disease during its cyclic interventions in the human body. We identified 488 genes that were differentially expressed at various stages of TB and allocated to pathways and gene set enrichment analysis. These pathways as well as GSEA’s importance were evaluated according to the number of DEGs presents in both. In addition, we studied the gene regulatory networks that may help to further understand the molecular mechanism of immune response against the TB infection and provide us a new angle for future biomarker and therapeutic targets. In this study, we identified 26 leading hubs which are deeply rooted from top to bottom in the gene regulatory network and work as the backbone of the network. These leading hubs contains 31 key regulator genes, of which 14 genes were up-regulated and 17 genes were down-regulated. The proposed approach is based on gene-expression profiling, and network analysis approaches predict some unknown TB-associated genes, which can be considered (or can be tested) as reliable candidates for further (*in vivo*/*in vitro*) studies.

## Introduction

Tuberculosis (TB) is a communicable disease generally caused by the bacterium *Mycobacterium tuberculosis* (MTB). The bacteria typically affects the lungs (pulmonary TB), but other body parts can be also affected (extra pulmonary TB) (Ahmad, 2011). The disease is communicable and spread through the air by expelling out the active MTB while coughing and sneezing (Schnappinger et al., 2003). In 2016, 10.4 million individuals were infected with TB, and 1.7 million died from the disease (including 0.4 million among people with HIV i.e., 40% of HIV deaths were due to TB). TB kills more adults in India than any other infectious disease (In 2016, an estimated 28 lakh cases occurred, and 4.5 lakh people died due to TB). India has the highest burden of both TB and advanced TB (like MDR TB) and second highest of HIV-associated TB. In India, the major challenge to curb the TB is poor primary healthcare system in rural areas due to deregulation of private health care leading to indiscriminate use of first- and second-line TB drugs, poverty, spreading HIV infection, and lack of administrative coordination among government functionary bodies. In our current study, we used meta-analysis of individual raw microarray data (GSE series) deposited in the GEO database which are obtained from various blood cell types (macrophages, monocytes, and CD4+) and cell lines (THP1) of individuals with different datasets (e.g., controls *vs*. TB disease, control *vs*. latent TB, latent TB *vs*. TB disease). We have performed gene-transition study of differentially expressed genes (DEGs) data and text mining between different stages of TB and classified the DEGs in stage-specific manner like normal to latent TB infections, normal to active TB, and latent infection to active TB. It is widely thought that, to know that function of a gene, it must be analyzed in the context of gene interaction network, because gene networks are commonly interpreted as encoding functional information in their connections. So, our study usually focused on the “guilt-by-association (GBA)” presumption which state that physically and functionally linked genes are possibly participating in the same biological pathways having comparable effects on the phenotypes (Lee et al., 2011). The concept of network theory is an imperative method to know the topological properties and the complex-system dynamics that correspond to their functional modules. The complex networks may be classified into four types of networks: (a) scale-free network, (b) small-world network, (c) random network, and (d) hierarchical network. For the biologist, hierarchical network has special interest because it incorporates the mien of modules, and distributed hubs (sparsely) regulate the network. So, we constructed the gene regulatory network and then analyzed its topological properties, because it helps to understand the structure of a network which facilitates in understanding the hidden mechanisms. Further, we identified important network modules which contain fundamental key regulators that have a fundamental importance.

## Material and Method

### Inclusion Criteria for Differentially Expressed Genes

A set of 11 microarray data sets were selected from the NCBI GEO repository database (http://www.ncbi.nlm.nih.gov/geo/). The datasets include GSE54992 (Cai et al., 2014), GSE52819 (Verway et al., 2013), GSE64335 (Bouttier et al., 2016), GSE11199 (Thuong et al., 2008), GSE98750 (Lavalett et al., 2017), GSE78233 (Cheng et al., 2017), GSE57736 (Guerra-Laso et al., 2015), GSE16250 (Reyes et al., 2015), GSE27882 (Liu et al., 2012), GSE57028 (Salamon et al., 2014), and GSE83456 (Blankley et al., 2016). The background data correction and data normalization were done by the robust multiarray average (RMA) in R affy and lumi packages to ensure unbiased and dysregulated gene expression data. The RMA method, which performs quantile normalization, was used to minimize inconsistencies due to normalization of the each Affymetrix GSE series. The RMA method was chosen over others due to its fine differential change detection and stable variance on a log scale and minimizes the false positive results. The specificity and sensitivity of RMA method are good during fold-change calculation to identify DEGs. Similarly, we have used lumi pipeline (Bioconductor package) which is exclusively developed to analyze Illumina data (BeadChip). It checks the data quality and data normalization and stabilizes the data variance. The gene expression data (GSE57736 and GSE27882) which is generated by Agilent platforms were already present as normalized data. In the present study, we used linear model for microarray analysis (LIMMA) package which is a highly recommended method to measure the differential expression of genes. It not only calculates simple t-test but also calculates moderate t-test and f-test by applying the *Empirical Bayes* approach and reducing the standard errors and gives us steady and reproducible outcomes even with a less quantity of arrays. So, limma package (Smyth, 2004) was used to identify the DEGs among the healthy control, latent TB, and active TB. We set the criteria to select significant differentially expressed genes i.e., the adjusted p-value was <0.05, and the fold change was >1.5. We have used the BRCW [Bioinformatics & Research Computing website (http://jura.wi.mit.edu/bioc/tools/compare.php)] to select the DEGs which is common in at least two datasets of gene expression profile. Doing this, we became more specific in the selection of DEGs and the chances of biased data compilation became negligible. According to the corresponding correlation between the probe and gene from the data, the probe numbers of the expression profile were changed into the corresponding gene symbols using the Synergizer database (Huang et al., 2009).

### Gene Classification, Ontology, and Pathway Analysis of Degs

To know the significance of the identified DEGs, we have categorized them by GO-molecular function, GO-biological process, and protein class using the Protein ANalysis THrough Evolutionary Relationships (PANTHER v.13.0) Classification System and analysis tools and DAVID database (https://david.ncifcrf.gov/) to enrich the given set of DEGs to possible GO terms (Mi et al., 2013) (Huang et al., 2009). The PANTHER overrepresentation study (Fisher’s exact with FDR multiple test correction) was used to search the data against the PANTHER, and GO databases and p-values were set according to Bonferroni correction.

The GO analysis (gene ontology) is the useful method for annotation of genes and its products and characterization of biological attributes for high-throughput genome or transcriptome data (Ashburner et al., 2000). The differentially expressed genes among “active-TB” and “latent TB” on compared with “normal condition” were over-represented in various GO classes. The gene ontology provides and visualizes us the basic terms subdivided into three important categories, namely, BP (Biological process), MF (molecular functions), and biological pathways among these groups.

### Gene Transition Among Different Stages of TB

In order to get the behavior of normal gene expression perturbation, we tried a normal way for finding the associated genes while moving from one stage to another. In all the transitions that we took into consideration have been provided by a list of up- and down-regulated genes. These gene(s) in both the cases (i.e., stage from which is transferred *to* the targeted stage) have its own meaning. This meaning to a gene(s) regulation gives us an opportunity to say something about the ongoing mechanobiology inside the cell. So, to observe these transitions, we framed our study in such a way (based on the data we’ve got) which considers every possible transition in view. These transitions are discussed in brief as follows. In this study, we made comparison of the gene-expression profiles among individuals with normal conditions, latent infection, and active TB. Thus, we observed the expression of genes from normal to different stages of the TB and try to arrest those genes which play a key role in susceptibility to TB.

- **Normal to Latent Infections:** In this section, we have taken those DEGs which are involved in between normal and latent TB conditions. In LTBI, the bacteria remain in inactive form for many years (years to decades) before transforming into TB disease. In this study, we have studied several BPs and important pathways that lead to the latent infection for identification of latently infected individuals.
- **Normal to Active TB:** In this section, we have taken those DEGs which are differentially expressed in TB disease condition as compared to healthy control and recognized those immune process and pathways which are prominent in TB disease.
- **Latent Infection to Active TB:** In this section, we have taken those DEGs which are involved in between latent TB and active TB diseases. The individual with LTBI eventually reactivates and becomes infectious, seriously influencing epidemiological situation. Mechanisms of MTB transition to dormancy and TB reactivation are inadequately understood, and biomarkers of latency remain largely mysterious (Kondratieva et al., 2014).

### Topological Characterization of Networks

The classified genes of various stages of TB were used to construct their regulatory networks. The networks were built by STRING database (https://string-db.org/) and then visualized in Cytoscape v3.4 (Shannon et al., 2003). As the network was built, the first and foremost basic analysis are its topological properties. Topological analysis helps to understand the structure of a network which facilitates in understanding the hidden mechanisms. The following networks properties were analyzed to seek the important behaviors of the network:

- ***Degree distribution:*** The degree (k) of a node (gene) in a biological network is the number of links with other nodes, the probability distribution of these degrees called degree distribution (P[k]).

(1) $$P\left(k\right)=\frac{n_{k}}{N}$$

where**n** **_k _**=no. of nodes with degree k.

**N**= the total number of nodes in the network.

- ***Neighborhood connectivity:*** A node (gene) has number of neighbors and is considered as a connectivity of node. So, *neighborhood connectivity (C* *_N_* *[K]) is:*

(2) $$C_{N}\left(k\right)=\sum_{q}qP\left(\frac{q}{k}\right)$$

where $P\left(\frac{q}{k}\right)$ = the conditional probability.

- ***Clustering co-efficient:*** The ratio of number of edges (**e** **_i_**) between the node’s neighbor or maximum numbers of edges that could possibly exist between the nodes. So, the total network cluster coefficient is the average of cluster coefficient of all nodes (**i** **^th^** ) in the network.

(3) $$C\left(k_{i}\right)=\frac{2e_{i}}{k_{i}\left(k_{i}-1\right)}$$

- ***Betweenness centrality:*** In the complex network, a node’s betweenness centrality (CB) represents the prominence of information flow through one node to another *via* shortest path (Brandes, 2001) (Mason and Verwoerd, 2007). From node (**i**) to node (**j**), the geodesic paths are shown by “**dij(v)**” passing *via* node “**v**” and “**dij**.”

(4) $$C_{B}\left(v\right)=\sum_{i,j,i\neq j\neq k}\frac{d_{ij\left(v\right)}}{d_{ij}}$$

- ***Closeness centrality:*** In the network, how quick info is circulated from one node (i) to another (j) is measured by closeness centrality (C_C_). Closeness centrality is given by:

(5) $$C_{C}\left(i\right)=\frac{n}{\sum_{j}dij}$$

- ***Eigenvector centrality:*** In the network, eigenvector centrality of a node “**i** **(C** **_E_** **[i])**” is proportionate to the total of **i**’s neighbor centralities (Bonacich, 1987).

(6) $$C_{E}\left(i\right)=\frac{1}{\lambda }\sum_{j=nn\left(i\right)}v_{j}$$

where **nn(i)** = closest neighbors of nodes (“**i**”).

λ = eigenvalue of the eigenvector.

**v** **_i_** = “**Av** **_i_** **=λv** **_i_**” where “**A**” (adjacency matrix). 

### Community Analysis: Leading Eigenvector Method

In hierarchical network, to distinguish the nature of modular and its properties is important to explain the activities of network at various levels of hierarchy. In our study, the leading eigenvector method (LEV) (Newman, 2006a; Newman, 2006b) was used to detect the communities in R from package “**igraph**” (Gabor and Nepusz, 2006) (the community detection script is given **Supplementary Data 3**).

- *Modularity*: Modularity determines to measure the strength of division of a network into clusters or communities (Newman and Girvan, 2004).

(7) $$Q=\frac{1}{2m}\sum_{ij}\left(A_{ij}-\frac{k_{i}k_{j}}{2m}\right)\delta \left(C_{i},C_{j}\right)$$

where **m** = **T**otal no. of edges.

**A** **_ij _**= adjacency matrix of size “**i** × **j**.”

**k** = degrees.

δ = function yields **1** if nodes “**i**” and “**j**” are in the same community.

### Genes Tracing

To access the regulation of network, we first tried to find out the most influential nodes (genes) within the network. The gene tracing (up to motif level) was done purely on the appearance of the respective genes in various submodules obtained from the clustering. Then, these genes were used to get the picture of changes in the network organization in their absence.

### Hub Gene Knockout

To know the changes of organization within the complex network in the absence of most influencing gene (node), we must remove constructed leading hubs (rich clubs) in the networks. We consecutively eliminated all the important hubs (one by one) from each network and measured the topological properties of this reorganized network to observe the regulating abilities of these hubs by calculating the degree of structural change due to their absence. 

### LCP-DP Approach to Estimate the Network Compactness

The LCP-decomposition-plot (LCP-DP) provides a way to characterize the topological properties of the network in 2D space of common neighbors (CN) index of connecting nodes and local community links (LCL) of each pair of interacting nodes in the network (Cannistraci et al., 2013). The LCP correlation defined by:

(8) $$LCP-corr=\frac{cov\left(CN,LCL\right)}{\sigma_{CN}\sigma_{LCL}}with\;CN>1$$

where *cov*(*CN*, *LCL*) = the covariance among *LCL* and *CN*.

σ**_LCL_** and σ**_CN_** = standard deviations.

(Note: MATLAB script is given in **Supplementary Data 2**).

### Hamiltonian Energy Estimation: Energy Distribution in the Network

The HE is used to organize a network at a certain level by following the formalism of *constant Potts model* (Traag et al., 2011; Traag, 2013). The energy distribution at the global and modular levels of the network is given by HE. HE of a network can be measured by:

(9) $$H^{\left[c\right]}=-\sum_{c}\left[e_{c}-\gamma n_{c\;}^{2}\right]$$

where **e** **_c_** = no. of edges.

**n** **_c_** = no. nodes.

 “**c**” and “γ” = the resolution parameter

## Results

### Differentially Expressed Genes (DEGs) Classification and Overrepresentation Analysis

**Figure 1:**
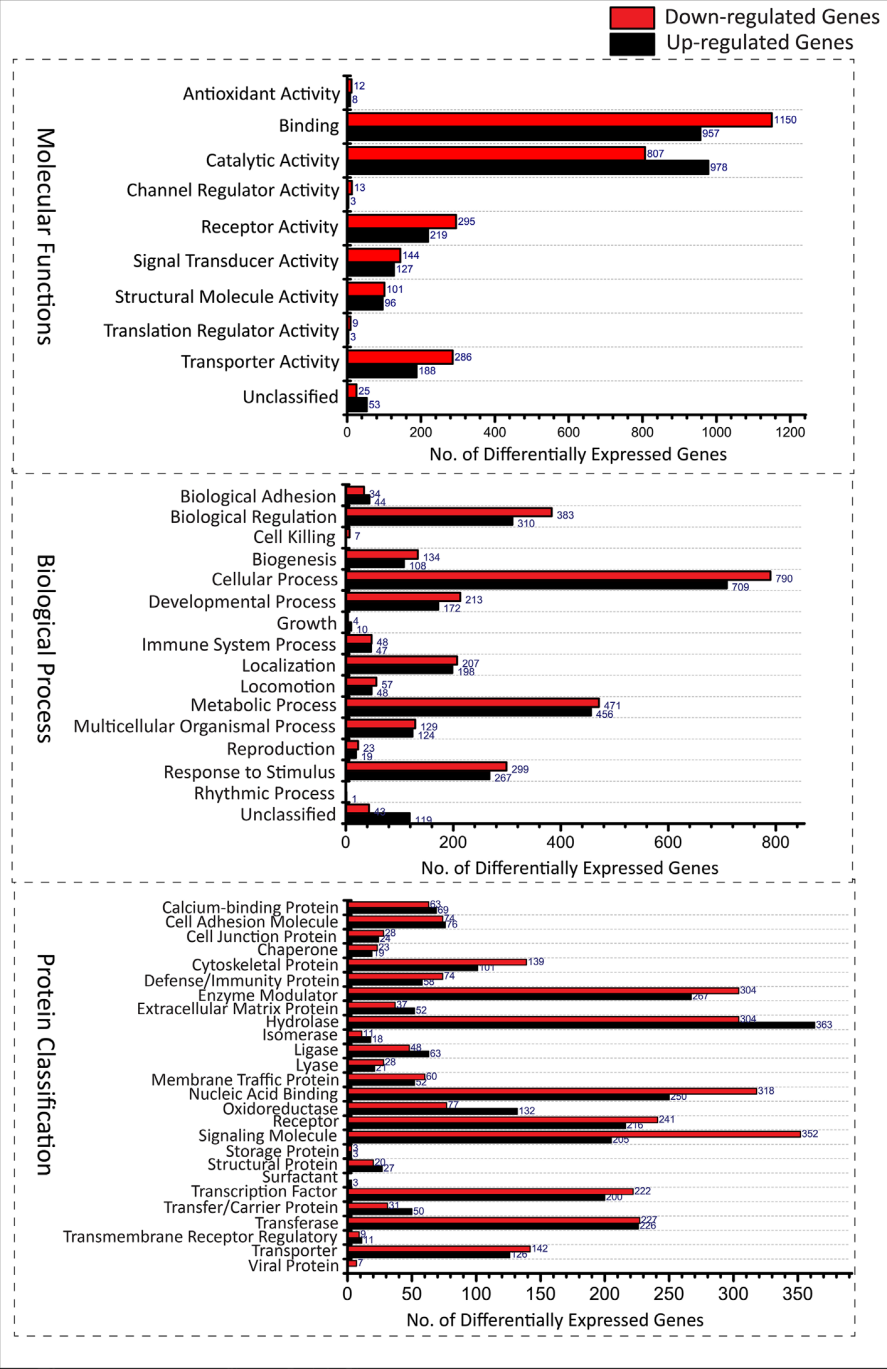
Functional Classification of Differentially expressed genes from various GSE series associated with TB disease according to **(A)** Molecular functions (Transporter activity [(U^188^,D_286_), Translation regulator activity(U^03^,D_09_), Catalytic activity(U^978^,D_807_), Channel regulator activity(U^03^,D_13_) Receptor activity(U^219^,D_295_), Signal transducer activity(U^127^,D_144_), Antioxidant activity(U^08^,D_12_), Structural molecule activity(U^96^,D_101_), Binding(U^957^,D_1150_) and unclassified(U^53^,D_25_)], **(B)** Biological processes [(cellular component organization or biogenesis(U^108^,D_134_), cellular process(U^709^,D_790_), localization(U^198^,D_207_), reproduction(U^19^,D_23_), biological regulation(U^310^,D_383_), response to stimulus(U^267^,D_299_), developmental process(U^172^,D_213_), rhythmic process(U^01^,D_00_), multicellular organismal process (U^124^,D_129_), locomotion(U^48^,D_57_), biological adhesion(U^44^,D_34_), metabolic process(U^456^,D_471_), growth(U^10^,D_04_), immune system process(U^47^,D_48_), cell killing (U^00^,D_07_) and unclassified(U^119^,D_43_)] and **(C)** Protein classes [(extracellular matrix protein(U^52^,D_34_), cytoskeletal protein(U^101^,D_139_), transporter(U^126^,D_142_), transmembrane receptor regulatory/adaptor protein(U^11^,D_09_), transferase(U^226^,D_227_), oxidoreductase(U^132^,D_77_), lyase(U^21^,D_28_), cell adhesion molecule(U^76^,D_74_), ligase(U^63^,D_48_), nucleic acid binding(U^250^,D_318_), signaling molecule(U^205^,D_352_), enzyme modulator(U^267^,D_304_),calcium-binding protein(U^69^,D_63_), defense/immunity protein(U^58^,D_74_), hydrolase(U^363^,D_304_), transfer/carrier protein(U^50^,D_31_), membrane traffic protein(U^52^,D_60_), transcription factor(U^200^,D_222_), chaperone(U^19^,D_23_), cell junction protein(U^24^,D_28_), surfactant(U^03^,D_00_), structural protein(U^27^,D_20_), storage protein(U^03^,D_03_), isomerase(U^18^,D_11_), receptor(U^216^,D_241_)]. (U, Up-regulated; D, Down-regulated).

A total of 5, 680 differentially expressed genes (DEGs) were identified after the extensive analysis of all the 11 GSE series, of which 2,660 were up-regulated, and 3,020 were down-regulated genes. These differentially expressed genes were clustered according to “GO-MF (molecular function),” “GO-BP (biological process),” and “PANTHER protein class” shown in **Figure 1**. All these predictive DEGs showed a broad range of protein classes which involved in various processes. The helicases and nucleases were found within the “nucleic acid binding” protein class. The “enzyme modulator” category features kinase, G-protein, phosphatase, and protease modulators. The structural motif and nuclear hormone receptors are part of the “transcription factor” protein class. The “hydrolases” is a sub-category of proteases and phosphatases. The “receptor” protein class includes cytokine receptors, protein-kinase receptors, ligand-gated ion channels, nuclear-hormone receptors, and G-protein-coupled receptors. Besides of these above protein classes, signaling molecules, transferase, oxidoreductase, and transporter are also most abundant protein classes. The two most abundant GO-biological process groups—“metabolic process” and “cellular process” which are not astonishing as these processes carry those genes which involved in the most basic life processes. The “cellular process” includes cell cycle, cell–cell signaling, cell component movement, cytokinesis, and proliferation. The “metabolic process” section includes metabolism of lipid, protein, carbohydrate, cellular amino acid, and nucleobase-containing compound metabolism. The “biological regulation” includes metabolism, cell cycle, the regulation of apoptosis, catalytic activity, translation, and homeostasis. Further, these biological process responses to stimulus, localization, and developmental process also represent the significant number of proteins; the complete categories details are given in **Supplementary Data 1**. To know the probability of largely occupied protein classes and GO categories among the differentially expressed genes, we used PANTHER’s overrepresentation analysis. When we compared with the reference genome, we found that any of the richest classes are overrepresented in the data (**Table 1**). The most abundant protein classes “chemokine,” “cytokine,” “hydrolase,” “ribosomal protein,” and “RNA-binding protein” were enriched along with the classes “signaling molecule” and “cell adhesion molecule.” The highly populated GO-biological processes were enriched in “cytokine-mediated signaling pathway,” “response to external stimulus,” “immune response,” “locomotion,” “signal transduction,” “cell communication,” “developmental process,” “cellular process,” and “MAPK cascade.” Similarly, the most abundant GO-molecular functions were enriched in “chemokine activity,” “cytokine activity,” “cytokine receptor binding,” “oxidoreductase activity,” and “receptor binding.”

**Table 1:** Overrepresented PANTHER protein class and GO ontology categories of all differentially expressed genes.

PANTHER Protein Classes	P-value	FDR
Chemokine	2.14E−08	2.29E−06
Cytokine	6.71E−08	4.79E−06
Hydrolase	5.82E−06	3.11E−04
Ribosomal protein	1.44E−05	6.15E−04
RNA-binding protein	2.36E−05	8.42E−04
Signaling molecule	1.46E−04	4.45E−03
Cell adhesion molecule	2.97E−04	7.95E−03
Protease	4.81E−04	1.14E−02
Receptor	1.52E−03	3.25E−02
**GO-Molecular Functions**		
Chemokine activity	1.19E−06	7.52E−05
Cytokine activity	1.18E−06	1.12E−04
Cytokine receptor binding	5.29E−05	1.67E−03
Oxidoreductase activity	4.85E−04	1.02E−02
Receptor binding	5.61E−04	1.07E−02
Hydrolase activity	3.87E−06	1.84E−04
Catalytic activity	9.50E−08	1.81E−05
Protein binding	1.20E−04	3.25E−03
Structural constituent of ribosome	1.66E−04	3.95E−03
**GO-Biological Processes**		
Cytokine-mediated signaling pathway	3.45E−08	4.21E−06
Response to external stimulus	3.75E−08	3.05E−06
Response to interferon gamma	1.65E−07	1.01E−05
Immune response	2.10E−07	1.02E−05
Sensory perception of chemical stimulus	2.15E−07	8.72E−06
Locomotion	3.38E−07	1.03E−05
Signal transduction	5.32E−07	1.44E−05
Immune system process	3.39E−06	8.27E−05
Cell communication	5.35E−06	1.19E−04
Cell proliferation	1.09E−05	2.23E−04
Response to biotic stimulus	1.59E−05	2.99E−04
Intracellular signal transduction	1.74E−05	3.03E−04
Death	4.17E−05	6.79E−04
Cell death	4.17E−05	6.36E−04
Cellular component movement	4.60E−05	6.61E−04
Developmental process	5.75E−05	7.80E−04
Apoptotic process	6.05E−05	7.77E−04
Cellular process	9.43E−05	1.15E−03
Sensory perception	9.74E−05	1.13E−03
Response to stress	1.04E−04	1.15E−03
RNA metabolic process	1.93E−04	2.04E−03
MAPK cascade	2.01E−04	2.05E−03
Response to stimulus	2.05E−04	2.00E−03
Cellular defense response	2.12E−04	1.99E−03
Endocytosis	3.17E−04	2.86E−03
Receptor-mediated endocytosis	7.23E−04	6.30E−03
Lipid metabolic process	9.30E−04	7.82E−03
Negative regulation of apoptotic process	1.01E−03	8.21E−03
Behavior	1.04E−03	8.15E−03
Localization	1.17E−03	8.90E−03
Regulation of catalytic activity	2.04E−03	1.51E−02
Sulfur compound metabolic process	2.06E−03	1.48E−02
Cell adhesion	2.78E−03	1.94E−02
Biological adhesion	2.78E−03	1.89E−02
Cell surface receptor signaling pathway	3.44E−03	2.27E−02
Cellular component biogenesis	3.88E−03	2.49E−02
Cell–cell adhesion	5.00E−03	3.13E−02
Cellular amino acid catabolic process	6.59E−03	4.02E−02
Regulation of molecular function	6.62E−03	3.94E−02
**GO-Cellular Components**		
Extracellular region	6.21E−05	1.96E−03
Extracellular space	5.48E−04	1.15E−02
Nucleus	2.01E−03	3.17E−02
Ribonucleoprotein complex	8.32E−06	5.24E−04
Nucleolus	3.28E−03	4.13E−02

Overrepresentation was determined by calculating the probability that the number of differentially expressed genes belonging to a category is larger or smaller than what would be expected based on a reference human genome. P-values are adjusted using a Bonferroni correction.

### Gene-Transition and GO Enrichment Analysis

**Figure 2:**
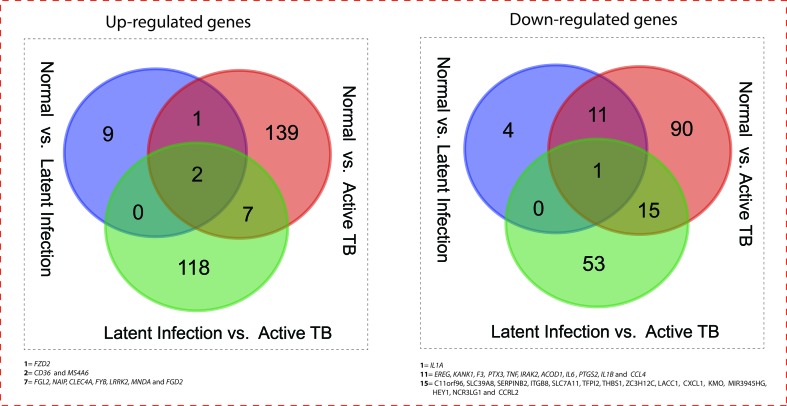
Venn diagram showing the number of common genes (in intersect region) which are differentially expressed among the normal *vs*. latent infection, normal *vs*. active TB, and latent infection *vs*. active TB.

All the 5,680 differentially expressed genes (DEGs) were divided into three different groups, normal *vs*. latent infection, normal *vs*. active TB, and latent infection *vs*. active TB. We filtered out a total number of 488 DEGs which are listed in (**Table 2**), and then, we also identified few genes which are commonly differentially expressed among various stages of TB (including NC *vs*. LI, NC *vs*. ATB, and LI *vs*. ATB) shown in **Figure 2**. Further, we isolated up- and down-regulated genes from each stage and performed GO-enrichment analysis using DAVID tool (v6.7). It is well preferred annotation database which used novel algorithms to extract biological information from large gene lists. The DEGs are enriched with a certain biological process or molecular function that are given in **Table 3**. Further, the most enriched pathways associated with these differentially expressed genes were identified, given in **Table 4**.

- **Normal to Latent infections:** In the early infection (latent infection), we have identified 28 genes which are differentially expressed in the host system, of which 12 genes (*IER5L, MS4A6A, DOK2, FZD2, NCKI-ASI, SNHG12, NLRC4, XPO7, SMA4, CD36, AFFI,* and *NDUFS8*) were up-regulated, and 16 genes (IL1A*, IL6, ACOD1, IL1B, ELOVL7, PTGS2, EREG, F3, IFIT1, TNF, KANK1, CCL4, CXCL11, PTX3, IRAK2,* and *AREG*) were down-regulated. The GO analysis of these DEGs are enriched in “immune response,” “inflammatory response,” “chemokine activity,” “cellular process,” “biological regulation,” “regulation of metabolic process,” “protein binding,” “catalytic activity,” “bindings” etc. On the pathway analysis, most of the DEGs were found to be down-regulated (beneficial to pathogen) and enriched in very important pathways like toll-like receptors, NF-kappaB signaling, cytokine–cytokine receptor interaction, MAPK signaling pathway, TB, and TNF signaling.
- **Normal to Active TB diseases:** A total of 266 differentially expressed genes (DEGs) were identified, of which 149 were up-regulated, and 117 were down-regulated among the various cases. In this condition, the DEGs were enriched in “inflammatory response,” “immune response,” “signal transduction,” “response to stimulus,” “apoptotic process,” “cellular process,” “metabolic process,” “binding,” “catalytic activity,” “receptor activity” etc. For the up-regulated genes, the most abundant pathways were “cytokine–cytokine receptor interaction,” “chemokine signaling,” “toll-like receptor signaling pathway,” “NF-kappa B signaling pathway,” “transcriptional misregulation in cancer,” and “pathways in cancer.” Similarly, for down-regulated genes, the most enriched pathways were “cell cycle,” “chemokine signaling pathway,” “NF-kappa B signaling pathway,” “TNF signaling pathway,” “PI3K-Akt signaling pathway,” “metabolic pathways,” and “MAPK signaling pathway.”
- **Latent infection to Active TB Disease::** A total of 127 differentially expressed genes (DEGs) were found to be up-regulated, and 69 were down-regulated. These differentially expressed genes were enriched in “binding,” “catalytic activity,” “receptor activity,” “transporter activity,” “cellular and metabolic process,” “biological regulation,” “immune response,” and “oxidoreductase activity” including others. The most abundant enriched pathway for up-regulated genes belonged to “metabolic pathway,” “NOD-like receptor signaling pathway,” “regulation of actin cytoskeleton,” “biosynthesis of antibiotics,” “metabolism of xenobiotics by cytochrome p450,” “thyroid hormone synthesis,” “regulation of autophagy,” and “peroxisome,” and for down-regulated genes, the enriched pathways were “chemokine signaling pathway,” “TNF signaling pathway,” “RAP1 signaling pathway,” “PI3K_AKT signaling,” “metabolic process,” “apoptotic,” “focal adhesion,” “MAPK signaling pathway,” “microRNAs in cancer,” and “amoebiases.”

**Table 2:** The total number of DEGs from various GSE series associated with TB disease.

Normal to Latent Infection	
Up-regulated	Down-regulated
IER5L, MS4A6A, DOK2, FZD2, NCKI-ASI, SNHG12, NLRC4, XPO7, SMA4, CD36, AFFI, NDUFS8	IL1A, IL6, ACOD1, IL1B, ELOVL7, PTGS2, EREG, F3, IFIT1, TNF, KANK1, CCL4, CXCL11, PTX3, IRAK2, AREG
Normal to Active TB	
Up-regulated	Down-regulated
GBP5, ISG15, SAMD9L, SERPING1, ANKRD22, ETV7, EPSTI1, GBP4, RSAD2, AIM2, IFI44, IFIT3, FGL2, FYB, MNDA, PAX5, OAS3, OAS1, IFI6, TNFSF10, UBE2L6, XAF1, STAT1, BST2, IFI35, STAT2, IFI44L, TRIM22, IFIH1, IFITM1, ATF3, BATF2, IFITM3, GBP1P1, RTP4, FCGR1B, C1QB, CEACAM1, FBXO6, SAMD4A, FRMD3, CMPK2, SELL, CFH, TLR8, LGALS3BP, SRGAP2, SECTM1, NCF1, SIGLEC1, APOL1, TRIM14, MB21D1, CARD16, FGD2, RNF213, CD163, PML, OAS2, OR52K3P, LY6E, RABGAP1L, P2RX7, NRG1, FBXO32, TYMP, PSMB9, NCF1C, PNPT1, CXCL13, GMPR, LAMP3, HESX1, C3AR1, STAT4, CXCL11, IFIT2, STAP1, ZC3H12A, RCAN1, HERC6, CCL20, CD83, CRIM1, GCH1, OASL, DLL4, MX2, EIF2AK2, EBI3, AXL, MGLL, CD80, IFIT5, IFIT1, CCL8, NFE2L3, PLSCR1, ICAM1, CXCL9, SLAMF7, CXCL10, HERC5, VCAM1, DDX58, NFKB1, SAMD9, IGFBP3, CCL3, CD274, BIRC3, IRF1, TAP1, PARP14, TMEM140, WARS, CASP1, GBP2, PSTPIP2, PARP9, RNASE6, FAM129C, FZD2, CD36, LRRK2, MS4A6A, CCR2, NAIP, FCRL2, P2RY13, CLEC7A, PCDH9, CD300LF, CLEC4A, C10orf54, BAIAP2-AS1, C1orf162, SORT1, JAK2, VAMP5, SCO2, ODF3B, PSME2, LOC101930164, P2RY14, GBP1, GBP6, CARD17, FCGR1A	RNF141, SLC25A37, ID3, EMP2, SKP2, SLC2A3, SUN1, KIT, OLR1, FLNB, CCDC14, GAPT, DHRS9, IQGAP3, SESN3, GINS4, HIST1H4C, CD44, KANK1, KCNJ2, TNIP3, MIR146A, CXCL3, LOC644090, MSC, SOD2, NLRP3, SERPINB9, TNFRSF9, ARL5B, IL24, ADORA2A, PHLDA1, MYO10, CXCL8, DNAAF1, MIR3945HG, NR3C1, TNFAIP6, SERPINB2, TNF, MAP3K8, IL1A, AGO2, CSF3, SPAG9, KYNU, LOC440934, WNT5A, DENND4A, ACOD1, PTGS2, OSM, CCL4, PFKFB3, EREG, ITGB8, PTX3, IL36G, G0S2, SLC7A11, ZC3H12C, TNFAIP3, IL6, CCRL2, FERMT2, SLCO4A1, SGPP2, FOSL2, CCL23, FLT1, SERPINB8, NUP98, SLC35F5, MN1, DDIT4, NAMPT, IRAK2, IL10, SLC7A5, AK4, CXCL2, UPB1, CEMIP, ADGRG2, FEZ1, THBS1, LACC1, CXCL1, TRAF1, PHLDA2, HEY1, LRP12, UBTD2, SLC39A8, PLPP3, SLC7A1, ATXN1, KMO, FNDC3B, IL1B, C11orf96, F3, PSEN1, BCAT1, GEM, TFPI2, PLAUR, MAFF, TRIM36, ZNF697, INSIG1, DPYSL3, ATP2B1, NCR3LG1, MAMLD1, ZNF540
Latent infection to Infection to Active TB	
Up-regulated	Down-regulated
CLEC12B, CD36, CORO1B, SIGLEC16, LINC00484, AK5, MS4A6A, GPBAR1, RTN1, CREB5, DPYD, LDB2, FCN1, LRRK2, RASSF4, ANXA4, LPCAT2, SKAP2, CPPED1, RNASE2, PLSCR3, CLEC12A, BST1, FGD2, RAB3D, FGL2, PYCARD, CEBPA, MNDA, CD33, PRAM1, LILRA1, SLC39A11, TNFSF13, SAMHD1, DIAPH2, FAR2, MSRB1, TBCK, FARS2, C14orf159, MSRB2, ATG16L2, DPYSL2, AIF1, HK3, SIGLEC7, FYB, RPS6KA4, TBC1D5, C10orf11, AGTRAP, PYGL, CARD9, NAGA, SLC9A9, C1RL, STX8, MTHFD1, KCTD12, CBR1, ASCL2, CPNE8, MBNL3, ANXA6, CALML4, HSDL2, SLC22A18, KDM1B, IDH1, DNAJC10, TBXAS1, SCLT1, HSD17B4, MGST2, NAIP, JAML, ENTPD1, ASGR1, BLVRB, AOAH, NIPAL2, NAAA, RAB24, TST, COMT, COMMD10, CYFIP1, TALDO1, ULK2, HDAC9, RBCK1, CEACAM4, OBFC1, FUCA2, NREP, STX10, AKR7A2, PLOD1, TRIOBP, QDPR, FAM172A, CDK19, DPAGT1, PARVG, CLEC4A, SSBP4, PNKP, FBXL5, ASRGL1, CARS2, ATP11A, PLXNC1, TSPO, ARHGEF6, AGPAT3, HEXDC, PDSS2, PGM2, PRKCB, H2AFY, S100A9, SNX15, NINJ2, MAP4, PSTPIP1, GSTK1	IL1A, ITGB8, TFPI2, COL1A1, MET, SERPINB2, GPRC5A, SLC7A11, CYP1A1, RAB7B, B3GALT2, DUSP4, CXCL1, MMP19, THBS1, HEY1, PPP1R10, ADAMDEC1, PDE4DIP, CCRL2, LMNA, ZC3H12C, RGS13, CXCL5, PMEPA1, SKIL, LACC1, DPYSL4, ABCG1, AHRR, MIR155, EPHA4, CD109, MIR3945HG, NCR3LG1, WHRN, FAM177A1, SLC39A8, HEY2, C11orf96, KMO, CYP1B1, DUSP16, NFKB1, LZTS3, CXCR3, PLIN2, BIRC3, DOCK4, EPB41L3, MYO5C, ZHX2, CD58, P2RY10, CD82, MAPK8, PARD6G, NAB1, ABCB4, AMPD3, STK38L, SPECC1L, IL10RB-AS1, BANP, ETV6, PDCD4, MPZL3, CASC7, GGA2

These genes are classified into various categories of TB like normal to latent infections that contain 28 genes, normal to active TB diseases that contain 265 genes, and latent infection to active TB that contains 195 genes in this category.

**Table 3:** Gene set enrichment analysis of differentially expressed genes (DEGs) among active TB, LTBI, and normal condition.

Normal to Latent Infection	Normal to Active TB	Latent Infection to Active TB
Immune response	Inflammatory response	Response to stimulus
Inflammatory response	Immune response	Biological regulation
Transcription factor activity	Signal transduction	Localization
Transferase	Apoptotic process	Cell adhesion
Transporter	GTPase activity	Protein phosphorylation
MAPK cascade	Biological regulation	Immune response
Chemokine activity	Localization	Oxidoreductase activity
Cellular process	Angiogenesis	Hydrolase activity
Biological regulation	Cell adhesion	DNA-templated
Response to stimulus	Cellular process	Angiogenesis
Metabolic process	Response to stimulus	Binding
Protein binding	Metabolic process	Catalytic activity
Catalytic activity	Binding	Receptor activity
Bindings	Catalytic activity	Transporter activity
Signaling molecule	Receptor activity	Structural molecular activity
		Signal transduction
		Cellular process
		Metabolic process

**Table 4:** Pathways enriched by differentially expressed genes (DEGs) among TB, LTBI, and healthy control (HC).

Normal to Latent Infection			
Up-regulated		Down-regulated	
Transcriptional misregulation in cancer	AFF1	Toll-like receptor	CCL4, CXCL11, IL1B, IRAK2, IL6, TNF
Phagosome, AMPK signaling pathway, ECM-receptor interaction, Hematopoietic cell lineage, PPAR signaling pathway, Adipocytokine signaling pathway	CD36	NF-Kappa B signaling pathway	CCL4, IL1B, PTGS2, TNF
		Cytokin–cytokin receptor interaction	CCL4, IL1A, IL1B, IL6, PTGS2
		MAPK signaling pathway	IL1A, IL1B, TNF
		Tuberculosis	IL1A, IL1B, IRAK2, IL6, TNF
Oxidative phosphorylation, non-alcoholic fatty liver disease (NAFLD, neurotrophin signaling pathway, Alzheimer’s disease	NDUFS8	TNF signaling	IL1B, IL6, PTGS2, TNF

NOD-like receptor signaling pathway	*NLRC4*			
Wnt signaling pathway	FZD2			
**Normal to Active TB**			
**Up-regulated**		**Down-regulated**	
Cytokine–cytokine receptor interaction	CCL3, CCL8, CCR2, CXCL10, CXCL11, CXCL13, CXCL9, TNFSF10, TNF	Cell cycle	SKP2, IQGAP3, BCAT1, ID3, IL10, MAPK3K8, PHLDA1, PTGS2, THBS1, TRIM36
Chemokine signaling pathway	CCL3, CCL8, CCR2, CXCL10, CXCL11, CXCL13, CXCL9, JAK2, NCF1, NFKB1, STAT1, TLR8, TNF	Chemokine signaling pathway	CCL20, CCL23, CCL4, CXCL1, CXCL2, CXCL3, CXCL8
Toll-like receptor signaling pathway	CCL3, CXCL10, CXCL11, CXCL9, CD80, BIRC3, ITF1, IRAK2, NFKB1, STAT1, TLR8, TNF	NF-kappa B signaling pathway	CCL4, CXCL8, TNFAIP3, TRAF1, IL1B, PTGS2
NF-kappa B signaling pathway	DDX58, BIRC3, ICAM1, NFKB1, TNF, VCAM1	TNF signaling pathway	CCL20, CXCL1, CXCL2, CXCL3, TNFAIP3, IL1B, IL6, MAP3K8, PTGS2
Transcriptional misregulation in cancer	ETV7, FCGR1A, IGFBP3, NFKB1, PAX5, PML	PI3K-Akt signaling pathway	DDIT4, KIT, CSF3, FLT1, ITGB8, IL6, OSM, THBS1
Pathways in cancer	BIRC3, FZD2, NFKB1, PML, STAT1	Metabolic pathways	AK4, UPB1, BCAT1, DHRS9, KYNU, KMO, NAMP, PLPP3, PTGS2
		MAPK signaling pathway	FLNB, IL1A, IL1B, MAP3K8
**Latent Infection to Active TB**			
**Up-regulated**		**Down-regulated**	
Metabolic pathway	AGPAT3, AK5, BST1, CBR1, COMPT, DPYD, DPAGT1, HK3, HSD17B4, IDH1, LPCAT2, MTHFD1, PGM2, PYGL, QDPR, TST, TBXAS1, TALDO1	Chemokine signaling pathway	CXCL1, CXCK5, CXCR3, NFKB1
NOD-like receptor signaling pathway	NAIP, PYCARD, CARD9, PSTPIP1	TNF signaling pathway	CXCL1, BIRC3, MAPK8, NFKB1
Regulation of actin cytoskeleton	ARHGEF6, TRIOBP, CYFIP1, DIAPH2	RAP1 signaling pathway	MET, DOCK4, PARD6G, THBS1
Biosynthesis of antibiotics	AK5, HK3, IDH1, PGM2, TALDO1	PI3K_AKT signaling	MET, COL1A1, ITGB8, NFKB1, THBS1
Metabolism of xenobiotics by cytochrome p450	AKR7A2, CBR1, GSTK1, MGST2	Metabolic process	AMPD3, B3GALT2, CYP1A1, KMO
Thyroid hormone synthesis	ASGR1, CREB5, PRKCB	Apoptotic	CXCR3, ETV6, SKIL, BIRC3, CYP1B1, EPB41L3, HEY2, IL1A, LMNA, MAPK8, NFKB1, PDCD4, SERPINB2, THBS1
Regulation of autophagy	PYCARD, TBC1D5, ATG16L2, LRRK2, ULK2	Focal adhesion	MET, BIRCC3, COL1A1, ITGB8, MAPK8, THBS1
Peroxisome	FAR2, GSTK1, HSD17B4, IDH1	MAPK signaling pathway	DUSPI6, DUSP4, IL1A, MAPK8, NFKB1
		MicroRNAs in cancer	MET, CYP1B1, MIT155, NFKB1, PDCD4, THBS1
		Amoebiases	RAB7B, COL1A1, NFKB1, SERDINB2

### Gene Interaction: Hierarchical Scale-Free Network

**Figure 3:**
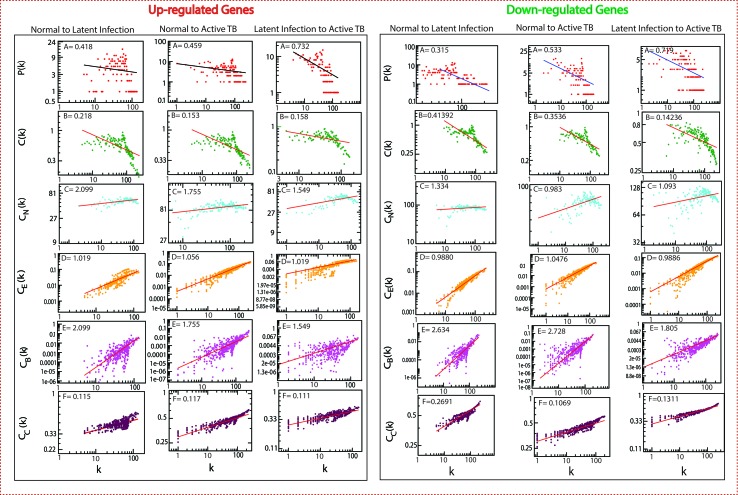
Topological properties of all the six networks. The behaviors of degree distributions (P[k]), clustering co-efficient (C[k]), neighborhood connectivity (C_N_[k]), betweenness (C_B_[k]), closeness (C_C_[k]), and eigenvector (C_E_[k]) measurements as a function of degree k. The lines are fitted lines with power laws in the data sets.

The classified genes of various stages of TB (**Table 2**) were used to construct their regulatory network. We constructed six networks for Up and Down-regulated genes separately. The topological parameters of the networks obey power law distributions. The probability of clustering co-efficient *C(k)*, degree distributions *P(k)*, and neighborhood connectivity *C* *_N_* *(k)* exhibits fractal nature as shown in **Figure 3**. The results for all the networks are summarized as follows:

$$\begin{array}{ll} \mathrm{Normal to latent Inf.} & \mathrm{Normal to active TB}\\ \left[\begin{array}{c} P\\ C\\ C_{N} \end{array}\right]∼\left[\begin{array}{c} k^{-A}\\ k^{-B}\\ k^{+C} \end{array}\right]\to \left[\begin{array}{c} 0.418\\ 0.218\\ 2.099 \end{array}\right] & \left[\begin{array}{c} P\\ C\\ C_{N} \end{array}\right]∼\left[\begin{array}{c} k^{-A}\\ k^{-B}\\ k^{+C} \end{array}\right]\to \left[\begin{array}{c} 0.459\\ 0.153\\ 1.755 \end{array}\right]\;\\ \left[\begin{array}{c} P\\ C\\ C_{N} \end{array}\right]∼\left[\begin{array}{c} k^{-A}\\ k^{-B}\\ k^{+C} \end{array}\right]\to \left[\begin{array}{c} 0.315\\ 0.413\\ 1.33 \end{array}\right] & \left[\begin{array}{c} P\\ C\\ C_{N} \end{array}\right]∼\left[\begin{array}{c} k^{-A}\\ k^{-B}\\ k^{+C} \end{array}\right]\to \left[\begin{array}{c} 0.533\\ 0.354\\ 0.983 \end{array}\right]\\ \mathrm{Latent Inf. to active TB} & \;\\ \left[\begin{array}{c} P\\ C\\ C_{N} \end{array}\right]∼\left[\begin{array}{c} k^{-A}\\ k^{-B}\\ k^{+C} \end{array}\right]\to \left[\begin{array}{c} 0.732\\ 0.158\\ 1.549 \end{array}\right] & \to \text{ For Up-regulated genes}\\ \left[\begin{array}{c} P\\ C\\ C_{N} \end{array}\right]∼\left[\begin{array}{c} k^{-A}\\ k^{-B}\\ k^{+C} \end{array}\right]\to \left[\begin{array}{c} 0.719\\ 0.563\\ 1.093 \end{array}\right] & \to \text{ For Down-regulated genes} \end{array}$$

Our network behavior indicates hierarchical scale free network (Barabási and Oltvai, 2004) (Pastor-Satorras et al., 2001) (Ravasz and Barabási, 2003). The power law fits on the data points of the network’s topological parameters were done and confirmed by following the standard statistical fitting method given by Clauset *et al*. (Clauset et al., 2009). The p values for all data sets were calculated (against 2,500 random samplings) and found to be greater than 0.1, and data fitting goodness was less than 0.1. The values of **P(k)** and **C(k)** were negative, which implies that the network follows a hierarchical pattern, and **C** **_N_** **(k)** was positive, which means that the network follows the assortativity that identifies a huge cluster of degree-nodes (rich club), which regulates the network. The centrality parameters: betweenness (C_B_), closeness (C_C_), and eigenvector (C_E_) centralities of the network also showed fractal behavior, and good connectivity of nodes in a network is distinguished by eigenvector or centrality C_E_(k). It calculates the effectiveness of the spreading (receiving) power of data of nodes from the network. These properties follow the power law behaviors as follows: -

$$\begin{array}{ll} \mathrm{Normal to latent Inf.} & \mathrm{Normal to active TB}\\ \left[\begin{array}{c} C_{B}\\ C_{C}\\ C_{E} \end{array}\right]∼\left[\begin{array}{c} k^{E}\\ k^{F}\\ k^{D} \end{array}\right]\to \left[\begin{array}{c} 2.099\\ 0.115\\ 1.019 \end{array}\right] & \left[\begin{array}{c} C_{B}\\ C_{C}\\ C_{E} \end{array}\right]∼\left[\begin{array}{c} k^{E}\\ k^{F}\\ k^{D} \end{array}\right]\to \left[\begin{array}{c} 1.755\\ 0.117\\ 1.056 \end{array}\right]\\ \left[\begin{array}{c} C_{B}\\ C_{C}\\ C_{E} \end{array}\right]∼\left[\begin{array}{c} k^{E}\\ k^{F}\\ k^{D} \end{array}\right]\to \left[\begin{array}{c} 2.634\\ 0.269\\ 0.989 \end{array}\right] & \left[\begin{array}{c} C_{B}\\ C_{C}\\ C_{E} \end{array}\right]∼\left[\begin{array}{c} k^{E}\\ k^{F}\\ k^{D} \end{array}\right]\to \left[\begin{array}{c} 2.728\\ 0.107\\ 1.048 \end{array}\right]\\ \mathrm{Latent Inf. to active TB} & \;\\ \left[\begin{array}{c} C_{B}\\ C_{C}\\ C_{E} \end{array}\right]∼\left[\begin{array}{c} k^{E}\\ k^{F}\\ k^{D} \end{array}\right]\to \left[\begin{array}{c} 1.549\\ 0.111\\ 1.021 \end{array}\right] & \to \text{ For Up-regulated genes}\\ \left[\begin{array}{c} C_{B}\\ C_{C}\\ C_{E} \end{array}\right]∼\left[\begin{array}{c} k^{E}\\ k^{F}\\ k^{D} \end{array}\right]\to \left[\begin{array}{c} 1.805\\ 0.131\\ 0.987 \end{array}\right] & \to \text{ For Down-regulated genes} \end{array}$$

### Identification of Key Regulators and Properties

**Figure 4:**
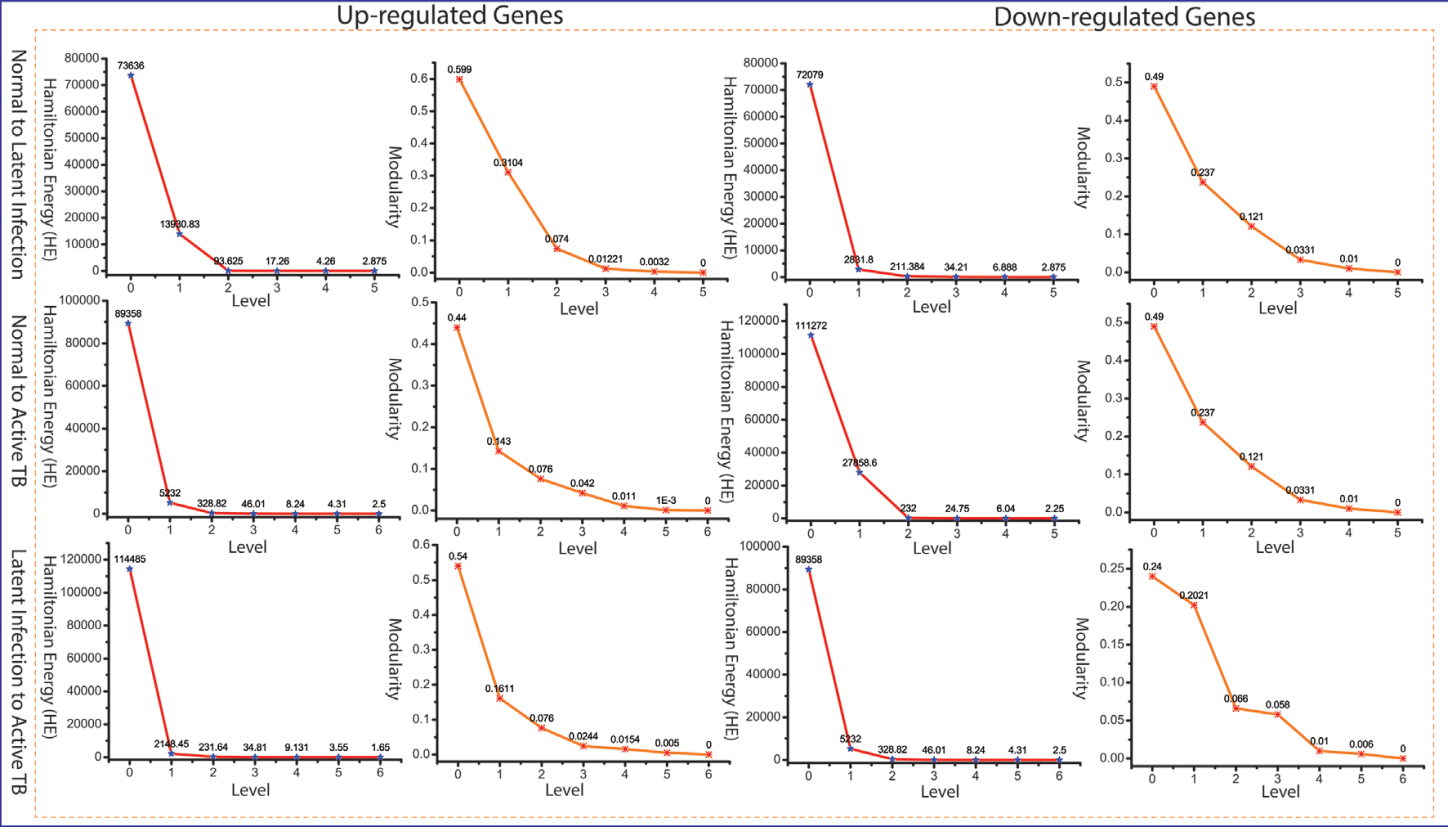
Energy and modularity distribution in all networks quantified by Hamiltonian (HE) and modularity calculation as a function of network levels.

Since the popularity of leading hubs gets changed according to the gene activities and its regulation, we can’t say that all the predictive leading hubs are key regulators for disease progression, but some of these hubs can play a significant role, which we called them as fundamental key regulators (FKR). The structure of modular and its arrangement have been performed through Newman and Girvan’s standard community finding algorithm at various levels of the organization. Using this community finding algorithm, we found that our six networks are hierarchically organized at various levels (**Supplementary Data 2**). The Hamiltonian energy (HE) and corresponding modularity (Q_N_) are reduced as one goes from top to down organization of network (**Figure 4**).

We have calculated the probability to know the regulating ability of our 31 key regulators:

$$P_{x}\left(y^{\left[l\right]}\right)=\frac{y^{\left[l\right]}}{E^{\left[l\right]}}$$

where,

**x** = no. of edges (***y*** ***^[l]^***)

(*E* *^[l]^*) = Total no. of edges of the network (modules and sub-modules).

**Figure 5:**
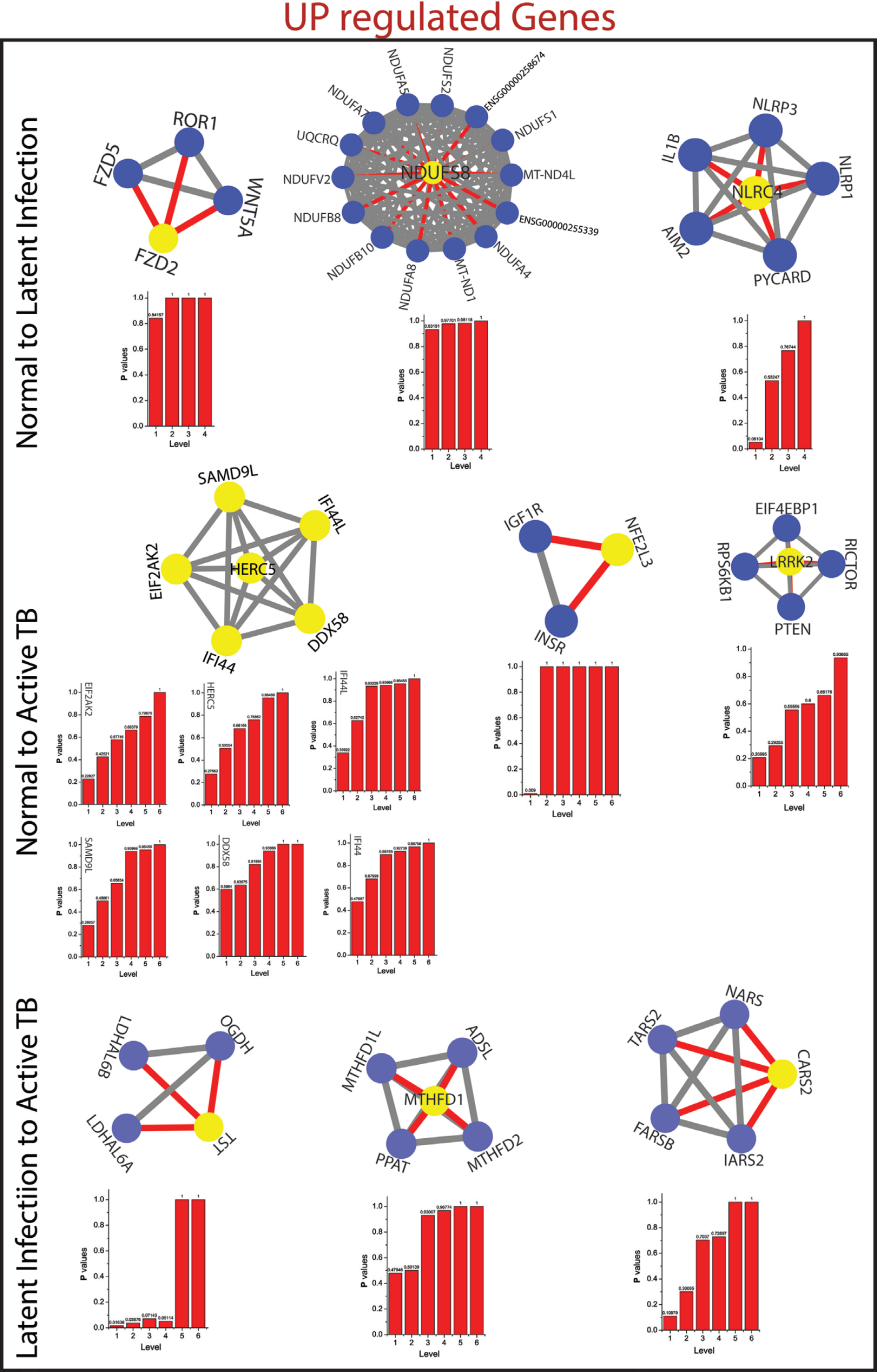
This figure shown the fundamental key regulator from various stage of TB obtained from main networks to motif/hub level through various modules/sub-modules at various level of organization. The probability distribution of the 14 up-regulated key genes as a function of level of organization.

**Figure 6:**
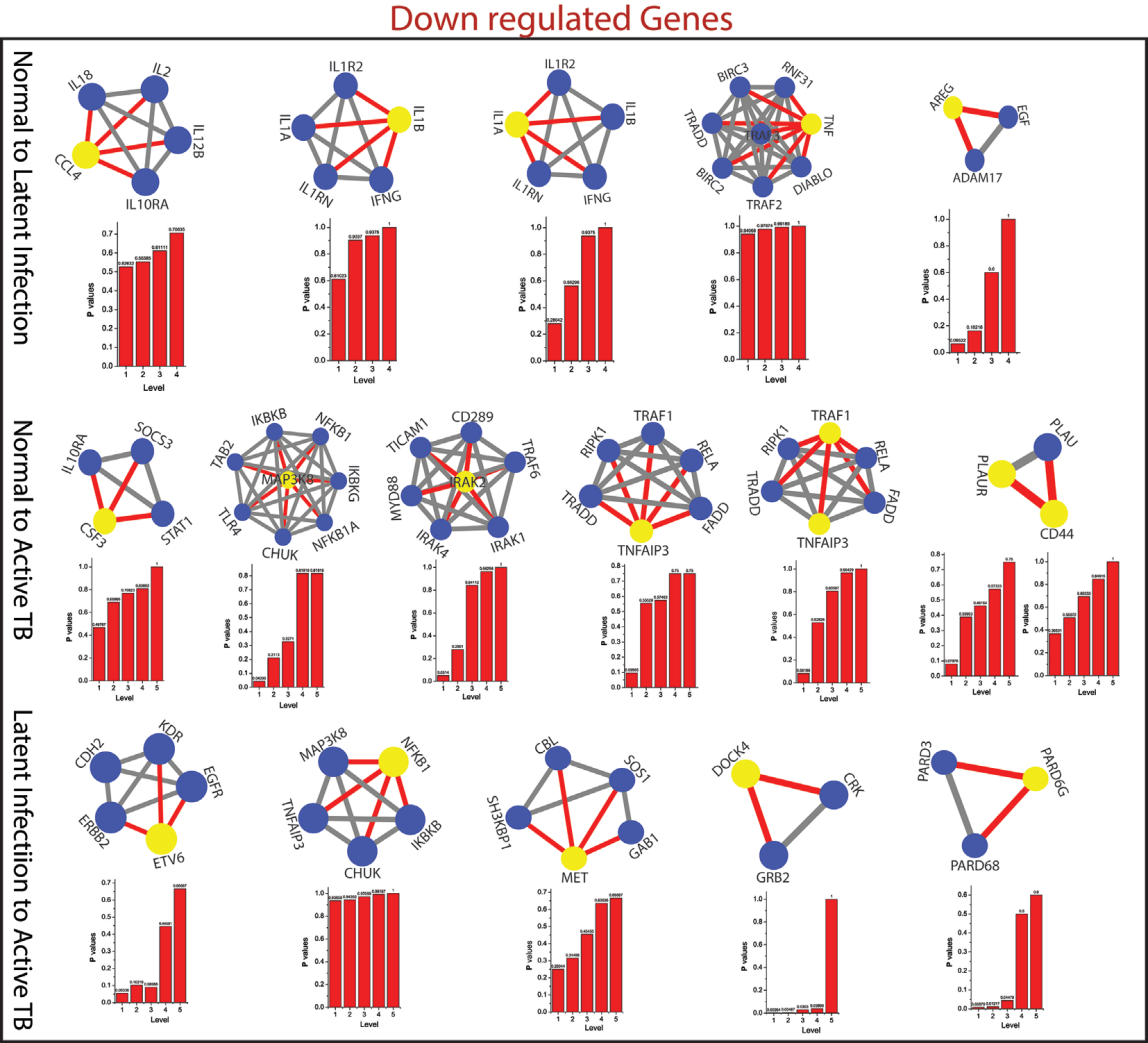
This figure shown the fundamental key regulator from various stage of TB obtained from main networks to motif/hub level through various modules/sub-modules at various level of organization. The probability distribution of the 15 down-regulated key genes as a function of level of organization.

The measured probability *P* *_x_*(*y* *^l^*) of all the KR showed an increase in P_x_ as the level the increases from top to bottom direction. At deeper level of the organization, regulation of FKR increases, and their activities become more prominent. Thus, these FKR becomes backbone of the network organization, stabilization, and active workers at grassroot level. The FKR are shown in **Figures 5** and **6**.

**Figure 7:**
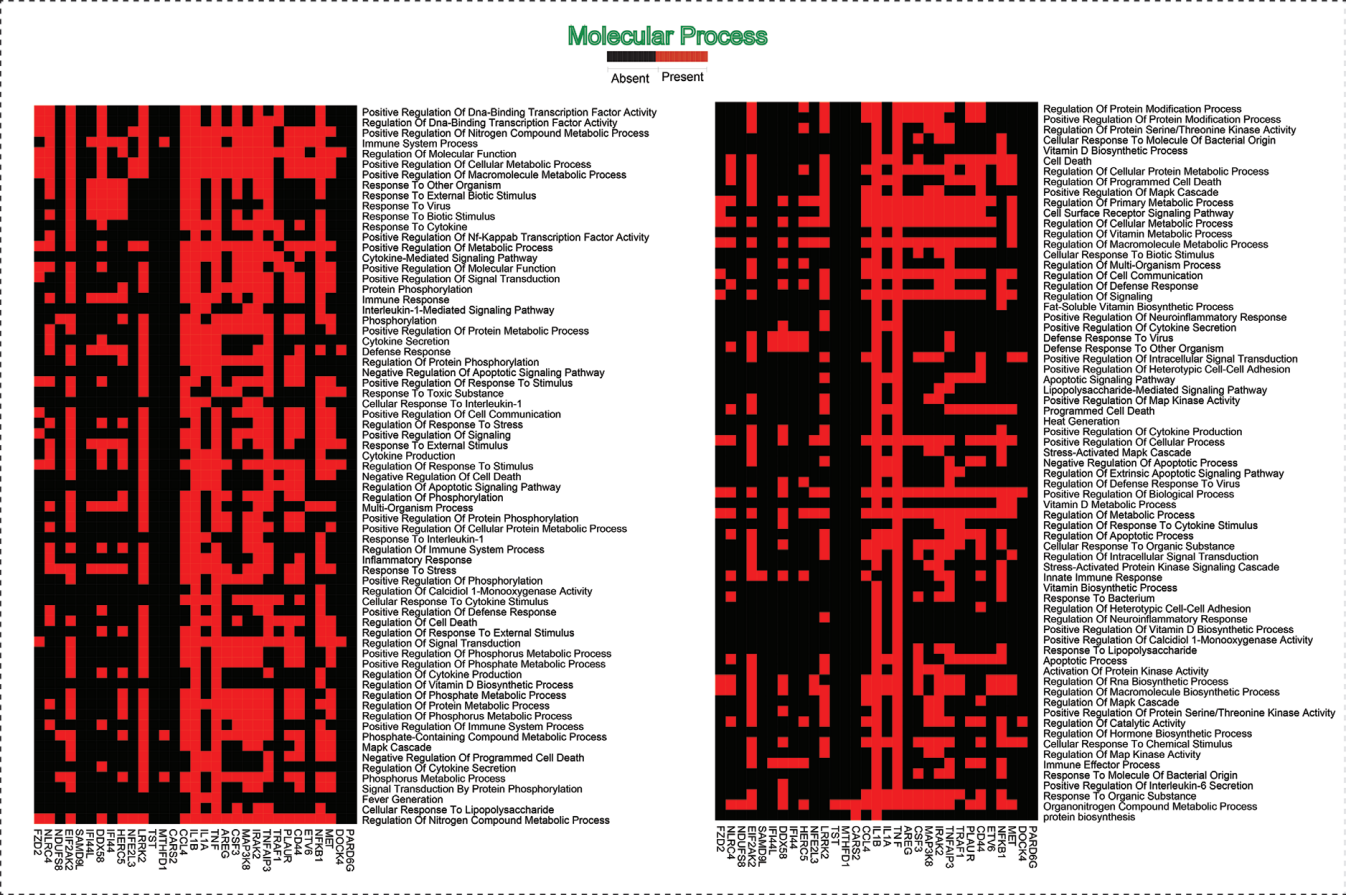
This heatmap shows the 31 hub genes with their involvement in various biological processes.

**Figure 8:**
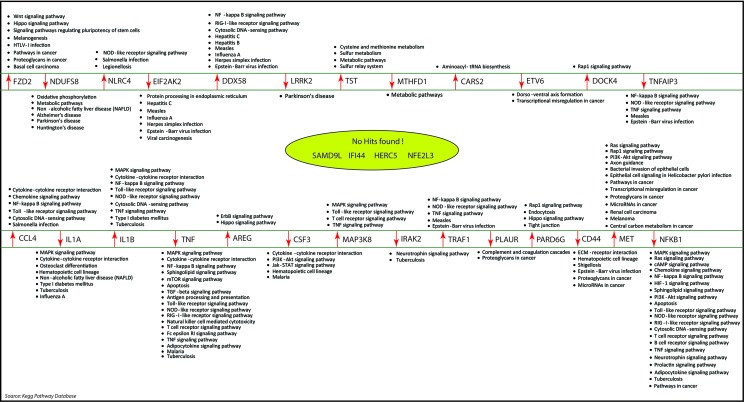
The molecular pathways associated with our key regulators were identiﬁed by KEGG pathway database. Out of the 31 key regulators, we did not find any pathways information of five genes namely: SAMD9L, IFI44L, IFI44, HERC5, and NF2L3.

As the results, we identified a total of 31 FKR genes from various stages of TB. In normal to latent infections, we identified eight genes, of which three genes (FZD2, NDUFS8, NLRC4) were up-regulated, and another five genes (CCL4, IL1B, IL1A, TNF, AREG) were down-regulated. While in normal to active TB, we found 15 genes out of which eight genes (EIF2AK2, SAMD9L, IFI44L, DDX58, IFI44, HERC5, NFE2L3, LRRK2) were up-regulated, and seven genes (CSF3, MAP3K8, IRAK2, TNFAIP3, TRAF1, PLAUR, and CD44) were down-regulated, and similarly, for latent to active TB diseases, three genes (TST, MTHFD1, CARS2) were up-regulated, and five genes (ETV6, NFKB1, MET, DOCK4, PARD6G) were down-regulated. The functional interpretations of these 31 key regulators were annotated by GO-TermFinder (LAGO) (Boyle et al., 2004) using biological process terms at P-value 0.01; the results are interpreted in **Figure 7**. The gene specific pathways were identiﬁed by KEGG pathway database (Kanehisa et al., 2016), and the results are shown in **Figure 8**.

### Local Perturbations of Network 

**Figure 9:**
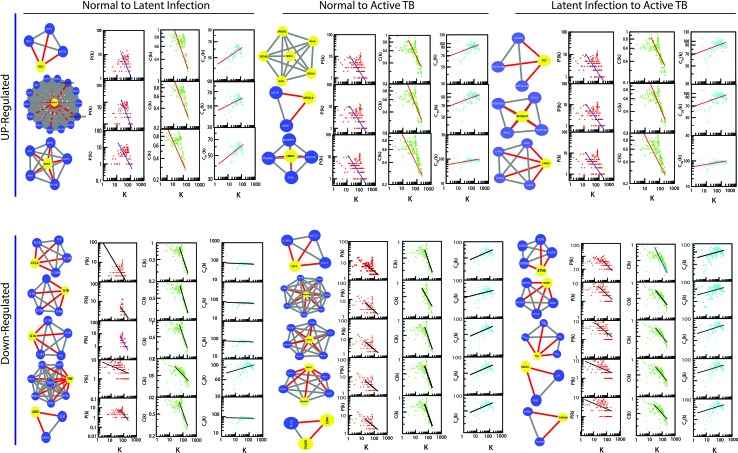
The changes in the exponents of the three important topological parameters (P[k]), C(k), and C_N_[k]) due to hub genes knock-out experiment from parent network.

The gene knockout experiment of all the hubs/motif from the parent networks may able to highlight the local perturbations driven by these individual hub or motif, and their effect on global network properties. It has been revealed that the network is tolerant to hub’s deletion which implies that the important network elements are still remains after elimination of hubs at level 0 (parent network). One of main causes is that the P-P interactions networks are too dense to be broken into fragments by only removing hubs. However, the elimination of these hubs/motif from the complete network does cause significant variations in the network properties, where **P(k)** and **C(k)** change significantly in complete network level, whereas **C** **_N_** **(k)** changes slightly. Likewise, the variations in the exponents of centrality measurements have also showed significant changes, as shown in **Figure 9**. Since, it is clear from the differences in the exponents of topological parameters that network perturbation increases when it goes to deeper level (top to down direction). In our case, most of the perturbation increases after the 3rd level; at this level elimination of key regulator from network almost breaks down the submodules existing in the deeper levels; such type of behavior shows that local perturbation of network is highest at deeper levels.

### The Local-Community-Paradigm: Evidence of Self-Organization

**Figure 10:**
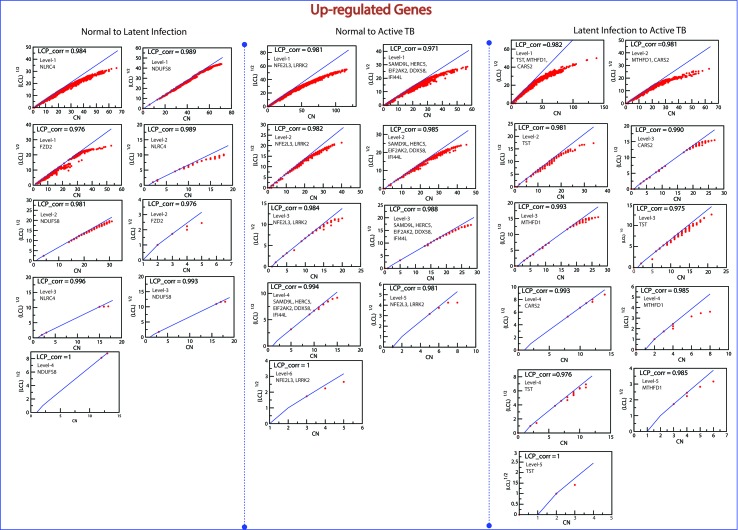
This figure shows the LCP-corr of all the modules/sub-modules of up-regulated genes at various levels. The compactness characterized by 2D plots between √(LCL) *versus* CA.

**Figure 11:**
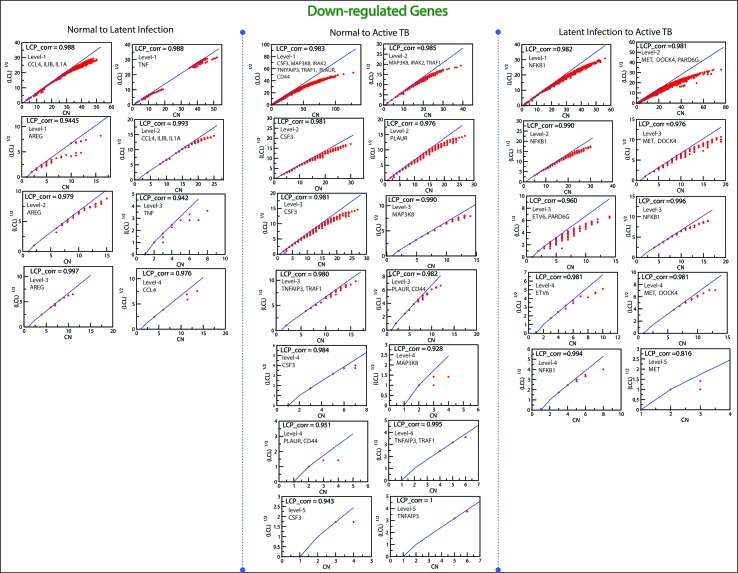
This figure shows the LCP-corr of all the modules/sub-modules of down-regulated genes at various levels. The compactness characterized by 2D plots between √(LCL) *versus* CA.

The LCP architecture supports the quick transfer of data across several network modules and through local processing too. We have analyzed all the six networks to check self-organization behavior at different levels using LCP method. For different level, the calculated LCP-corr of all the modules or sub-modules are shown in **Figures 10** and **11**. The average values of LCP-corr (we ignored modules which having zero LCP-corr) were greater than 0.853 at each level. This shows that the network maintains compactness and self-organization and has efficient data processing. It characterizes robust LCP networks that are dynamic in nature and heterogeneous, which help in network re-organization and evolution.

## Discussion

TB disease remains to be one of the most significant infectious diseases and a leading cause of death worldwide. Currently, the main challenge is to develop a delicate and effective method to identify the latent TB infection (LTBI) because 90% of cases of latent infection with MTB do not show any symptoms/signs but have a 10% lifetime possibility of transforming into active TB. As we know, many events have happened in response to TB infection cycle. According to the cycle of infection by Young et al., 2008, the exposure of microorganism up to its development to latent stage, there are stringent amount of processes that have appeared in the host cell. The long fight of MTB to establish its generation by stabilizing its environment for metabolism has evolved with an ability to overcome immunity. In this scenario of transferring from initial infection stage to latent stage has a profound increase in the overall immune response outside the host cell and an increased intensity of basic cellular machine establishment process for the pathogen cell. In case, the elimination eliminates the eliminator even after the induction of T-cell but able to suppress the effect; pathogen cells (Pcells) have two choices, either develop army or attack the adaption of immunity. In order to decide to enter the resting phase (i.e., latent), MTB’s survival instincts allow it to develop its metabolic environment by downregulating antigen presentation and the release of cytokines too (like IFNγ). On the other hand, if adaptive immunity has taken its chance, then MTB takes the defensive mode by subverting various normal cell cycle functioning inside the macrophages thus making a halt on the maturation of phagosomes. 

To stop the disease epidemic, early diagnostic method or techniques are required. In this way, the gene expression profiling has uncovered the differences in the transcriptome among normal condition, latent infection, and active TB. These results not only revealed significant genetic biomarkers indicative of LTBI, TB-disease conditions but also recognized transcriptionally regulated genes that vary in biological functions. In the current study, we have identified differentially expressed genes (DEGs) from various stages of disease and found that most of the DEGs relative to normal *vs*. TB disease condition (including latent infection) are enriched in important pathway like-toll like receptor, NOD-like receptor signaling pathway, MAPK signaling pathway, TNF signaling, chemokine signaling pathway, PI3K-Akt signaling pathway, apoptosis etc. In fact, pathogen is identified by the receptors on the surface of immune cells, and toll-like receptors (TLRs) are one of them. Different TLRs including TLR2, 4, 9, and 8 play an important roles in TB infection (Faridgohar and Nikoueinejad, 2017). These receptors (toll-like, NOD-like etc.) are expressed irrespective of whether they participate in immune signaling or immunity. The interaction of MTB with these receptors (like TLRs) initiates an intercellular signaling cascade that culminates in a pro-inflammatory response (cytokines and chemokines that serve as a signal for infection). As we know that MAPK signaling pathway is important for transcription and non-transcription responses of the immune system, most of the pathogens during infection hijack the immune system by targeting the MAPK signaling pathway (Soares-Silva et al., 2016). The TNF signaling pathway is involved in the regulation of immune cells, and it can regulate immune responses (Kim, 2018). However, pathogen can target the TNF signaling pathway for its survival in the host cell. The PI3K-Akt signaling pathway plays an important role in apoptosis, autophagy, metabolism, cell growth, and differentiation. The expression of FoxP3 by inhibiting the activation of transcription factor Forkhead-O3a (Foxo1-3a) is negatively regulated by this pathway (Zhang et al., 2017). The FoxP3+Treg cell activation which will assist to set up a new target for the involvement of TB immunotherapy molecules as part of the immune-escape mechanism to provide a theoretical basis is inhibited by *M. tuberculosis* (Scott-Browne et al., 2007). On comparing between latent infection and TB disease, few additional pathways are involved, like regulation of actin cytoskeleton during MTB infection; to maintain the stability of the cytoskeleton, macrophages cells themselves are also trying to regulate cytoskeletal-associated proteins (Wang et al., 2015b). Thyroid hormones (hormones, T4 and T3) are produced by thyroid gland, which is essential for the regulation of metabolic processes throughout the body. The regulation of autophagy is important for host in response to invading mycobacteria; the host defense mechanism identifies pathogen motifs through innate receptors but also releases appropriate cytokines. The autophagic pathways are regulated by these innate signals by regulation of genes of this pathway during infection (Jo, 2013). Recently, a study reports that the enzyme Msm_ACTase (from bacteria) helps in scavenging increased amount of H_2_O_2_ due to upregulation of genes involved in peroxisome pathway which gives an insight into a new idea as to how the MTB bacteria surpasses the host defense in MTB infection (Ganguli, 2015). Moreover, macrophages are the main effector cells responsible for killing pathogen (MTB) *via* different mechanisms, including apoptosis. But important genes in this pathway were down-regulated by MTB that slow down or stop the apoptotic process for its survival in host system (Behler et al., 2015). Most of the microRNAs that were found deregulated in cancer were affected by MTB in a similar way as its infestations, like deletion, mutation, and epigenetic silencing (Garzon et al., 2009). The apoptotic pathways are important to kill the infected macrophage. During MTB infection, the pathogen try to control the timing and mode of host cell death for its persistence and replication (Schaaf et al., 2017)

In addition, we have built the networks which emphasize on genes that were regulated by network. The constructed network of classified genes from various stages of TB showed hierarchical nature, which indicated that the networks have system level organization including modules/sub-modules which are interconnected. Since the network’s nature is hierarchical, its synchronization confirms several important functional regulations of the network, but individual gene activities are not so important. In our networks (including up- and down-regulated genes), a total of 31 key regulators were identified by affecting motifs and module regulation, showing their biological importance and serve as the foundation of network activities and their regulations, and could be the most probable target gene for disease control. We performed extensive analysis of all the key regulators (31 genes) to find evidence of their association with TB or which are directly or indirectly involved in host immune response and confirmed by manually searching for evidence in the literatures as presented:

- **Down-regulated genes:** We found that *TNF* and *IL1 (A and B)* are key mediators present in severe inflammatory diseases. However, both *IL-1* and TNF receptor pathways are important for the control of MTB infection, and it is critical to assess the respective role of *IL1A, IL1B*, and *TNF* (Bourigault et al., 2013) (Cavalcanti et al., 2012). The expression of *CCL4* is high in the late phase of the active TB disease, but there are low levels of expression during early infection (Rangel-Santiago et al., 2016). It has been currently reported that *AREG* plays an important role in orchestrating both host resistance and tolerance mechanisms. Although *AREG* is known as epithelial cell-derived factor, the recent studies showed that *AREG* can be expressed by multiple populations of activated immune cells in inflammatory conditions (Zaiss et al., 2015). The *NFKB1* activates the immune cells by up-regulating the expression of various cytokines in the host immune system (Ghosh et al., 1998). The gene *CD44* plays an important role in innate and adaptive immune responses, in the acute inflammatory response to both infectious and sterile stimuli. Also during infection, *CD44* may influence host defense by affecting phagocytosis (van der Windt et al., 2010). In our present study, we found that *NFKB1* and *CD44* are down-regulated which means that MTB is burglarized and seizing the host immune system by slowing down the expression of *NFKB1*. It is well known that MET (hepatocyte growth factor receptor) regulates several functions of immune cells, including cytokine production, differentiation and maturation, cellular migration and adhesion, and T-cell effector function, and HGF exerts anti-inflammatory activities through MET signaling (Sagi and Hieronymus, 2018). A group of researchers has elucidated that *PLAUR* domain containing Lypd8 that inhibits bacterial invasion of colonic epithelia (Okumura et al., 2016). The gene *TRFA1* has an active or passive interaction with many tumor necrosis factor receptor (TNFR), and it has been also shown that *TRAF1* plays as an antiapoptotic role in lymphoma cells by activation of NFKB (Wan et al., 2016). Further, the gene *TNFAIP3 (A20)* is a cytoplasmic zinc-finger protein which works as a negative-feedback regulator of NFKB activation (Vereecke et al., 2011). It has been shown that *IRAK2* is an important factor and potential novel biomarkers for human antiviral innate immunity (Wang et al., 2015a). Also, *MAP3K8* is a serine-threonine kinase and has a critical function in integrating host immune responses to complex pathogens (Mielke et al., 2009). Similarly, S. Srijata, *et al*. has suggested that AgNP attenuate the NF-κB pathway as indicated by the downregulation of NF-κB target genes *CSF3* and subsequently inhibit MTB-induced proinflammatory responses (Sarkar et al., 2015). The downregulation of these genes has allowed the process of host defense to cease and give time to bacteria for progression.
- **Up-regulated genes:** We found that *NLRC4* gene is associated with inflammasome signaling. Its upregulation means inflammasome activation (play an important role in host defense against MTB) not only leads to cytokine secretion but may also cause pyroptosis (Ting et al., 2008) (Bergsbaken et al., 2009). The *NDUFS8* genes also play an important role in host immunity by increasing the expression level (Lohman et al., 2017). The gene *FZD2* (frizzled class receptor 2) is Wnt pathway component (Wnt signaling), and Wnt signaling pathway plays a key role at different stages of TB development (Villaseñor et al., 2017). It has been reported that *LRRK2* is involved in the IFN-γ response and host response to pathogens (Gardet et al., 2010), and *LRRK2* also inhibits the immune response transcription factor NFAT1 (Liu et al., 2011). The genes *DDX58* interact with IRGM and promote its K63-linked polyubiquitination, indicating that IRGM is positioned at a nexus of various innate immunity (Chauhan et al., 2016) while SAMD9L and *EIF2AK2* play key roles in the innate immune responses to multiple stimuli. The gene *EIF2AK2* is also involved in the regulation of signal transduction, apoptosis, cell proliferation, and differentiation (Lemos de Matos et al., 2013) (Onomoto et al., 2012) (Reineke and Lloyd, 2015). It is well known that type-I IFN induces potent cellular defense against viral infection mediated by up-regulation of ISGs like *IFI44, IFI44L*, and *HERC5* (Jeon et al., 2010; Power et al., 2015). In our study, the expression of IFI44 and *HERC5* were found to be up-regulated that means host system tried to resist bacterial infection.

We didn’t find any literature evidence that supports the role of four genes (*NFE2L3*, *TST*, *MTHFD1*, and *CARS2*) and three genes (*ETV6*, *DOCK4*, and *PARD6G*) which are down-regulated and up-regulated respectively in host immune response against the pathogen, but most of these genes are involved in various types of cancers like *ETV6* is involved in prostate cancer as well as colorectal cancer susceptibility (Fararjeh and Liu, 2019; Wang et al., 2016); mutation in *DOCK4* can cause ovarian, prostate, glioma, and colorectal cancers (Sundaravel et al., 2015), and *PARD6G* suppresses cell proliferation and is targeted by loss-of-function mutations in several cancers (Marques et al., 2016). Besides of these *NFE2L3* (transcription factor) which involved in several cellular processes like inflammation, stress response, differentiation, and carcinogenesis (Kannan et al., 2015), *MTHFD1* are key players in folate metabolism, which is essential for *de novo* purine synthesis, and several defects in this pathway have been associated with immunodeficiency (Keller et al., 2013), and *CARS2* is associated with a severe progressive myoclonic epilepsy most resembling MERRF syndrome (Hallmann et al., 2014). The *TST* gene is a mitochondrial matrix enzyme (Pallini et al., 1991). It may play roles in cyanide detoxification, the formation of iron–sulfur proteins, and the modification of sulfur-containing enzymes.

## Conclusion

In this study, we have compared gene-expression profiling of active TB patient and latent TB patients, as well as healthy controls. We have combined different data sets from multiple microarray experiment, and this combined effect size method for the gene expression analysis gave us more biologically consistent and conservative results. We have identified differentially expressed genes and their associated particular biological processes and pathways among healthy controls, active TB and LTBI. The gene interaction networks employed to obtain functional modules and also uncovered novel key regulators for active TB and LTBI which might play an important role in regulating the expression of multiple TB-related host factors. In conclusion, our study provides new insights into host genes and pathways important for TB infection. Experimental validation of these hypotheses would confirm the credibility of the key regulators and make easier the development of a cost-effective and sensitive molecular diagnostic platform for TB disease.

## Data Availability Statement

Publicly available datasets were analyzed in this study. This data can be found here: https://www.ncbi.nlm.nih.gov/gds

## Author Contributions

RI conceived the study design instructed on data analysis. AA curated data and performed statistical and network analyses. NI, MM and AF implemented key statistical tools. AA, NI, MA, ST, AF, and NT drafted the manuscript. All the authors read, edited and approved of the manuscript.

## Funding

This work was supported by the Department of Health Research (DHR), Ministry of Health and Family Welfare, Government of India. Under the scheme of “Young Scientist” fellowship (R.12014/06/2019-HR).

## Conflict of Interest

The authors declare that the research was conducted in the absence of any commercial or financial relationships that could be construed as a potential conflict of interest.

## Abbreviations

MTB, *Mycobacterium tuberculosis*; DEGS, Differentially expressed genes; KEGG, Kyoto Encyclopedia of Genes and Genomes; GO, Gene Ontology; LCP, Local–community–paradigm; MDR, Multidrug-resistance TB; GBA, Guilt by association; MF, Molecular function; BP, Biological process; PC, Protein class; NC, Normal control; LI, Latent infection; ATB, Active TB; FKR, Fundamental key regulators; LAGO, Logically accelerated GO; GEO, Gene Expression Omnibus; GSE, GEO series; RMA, Robust multiarray average; LIMMA, Linear model for microarray analysis; CN, Common neighbors; HE, Hamiltonian energy; CPM, Constant Potts model; LCP-DP, LCP-decomposition-plot.

## Supplementary Material

Supplementary Data 1 Functional Classification of Differentially expressed according to (A) Molecular functions, (B) Biological processes and (C) Protein classes.

Supplementary Data 2 The modular path of important hub genes from complete network to motif level with the structures of modules/sub-modules at various levels.

Supplementary Data 3 (A) Community Finding (Network cluster Analysis) script in R. (B) LCP-correlation and LCP-decomposition-plot (LCP-DP approach to estimate the network compactness) method and MATLAB commonds.
