# Supplementary material for: Identification and Classification of Differentially Expressed Genes and Network Meta-Analysis Reveals Potential Molecular Signatures Associated With Tuberculosis
Source: Front Genet. 2019 Nov 4;10:932. doi: 10.3389/fgene.2019.00932 (PMC6844239; doi:10.3389/fgene.2019.00932)
Supplement: Supplementary Data 1 — Functional Classification of Differentially expressed according to (A) Molecular functions, (B) Biological processes and (C) Protein classes. [file DataSheet_1.pdf]

Functional Classification of Differentially expressed genes according to Molecular function (A), Biological process(B) and protein classes.

Molecular functions

1. Binding
- Amide binding
- Carbohydrate derivative binding
- Chromatin binding
- Cofactor binding
- Drug binding
- Heterocyclic compound binding
- Ion binding
- Lipid binding
- Molecular adaptor activity
- Organic cyclic compound binding
- Protein binding
- Protein-containing complex binding
- Small molecule binding

2. Catalytic activity
- Catalytic activity, acting on DNA
- Catalytic activity, acting on RNA
- Catalytic activity, acting on a protein
- Hydrolase activity
- Intramolecular transferase activity
- Ligase activity
- Lyase activity
- Oxidoreductase activity
- Transferase activity

3. Translation regulator activity
- DNA-binding transcription factor activity
- Transcription coregulator activity

4. Transporter activity
- Lipid transporter activity
- Transmembrane transporter activity

5. Structural molecular activity
- Extracellular matrix structural constituent
- Protein-containing complex scaffold activity
- Structural constituent of nuclear pore

6. Channel regulator Activity
7.Antioxident activity
8.Receptor activity
9. Signal transducer activity

Biological process

1. Cellular process
- Cell communication
- Cell cycle process
- Cell cycle
- Cell death
- Cell division
- Cellular component organization
- Cellular developmental process
- Cellular homeostasis
- Cellular metabolic process
- Cellular response to stimulus
- Establishment or maintenance of cell polarity
- Execution phase of apoptosis
- Gene silencing
- Microtubule-based process
- Movement of cell or subcellular component
- Secretion by cell signal transduction

2. Localization
- Cellular localization
- Establishment of localization
- Localization of cell
- Macromolecule localization
- Maintenance of location
- Protein-containing complex localization

3. Biological regulation
- Regulation of biological process
- Regulation of biological quality
- Regulation of molecular function

4. Response to stimulus
- Cellular response to stimulus
- Detection of stimulus
- Immune response
- Response to biotic stimulus
- Response to chemical
- Response to endogenous stimulus
- Response to external stimulus
- Response to stress

5. Developmental process
- Anatomical structure development
- Anatomical structure morphogenesis
- Cellular developmental process
- Developmental growth

6. Rhythmic process
- Circadian rhythm

7. Multicellular organismal process
- Cytokine production
- Multicellular organism development
- Ossification
- Pattern specification process
- System process

8. Biological adhesion
- Cell adhesion

9. Metabolic process
- Biosynthetic process
- Catabolic process
- Cellular metabolic process
- Nitrogen compound metabolic process
- Organic substance metabolic process
- Oxidation-reduction process
- Pigment metabolic process
- Primary metabolic process
- Small molecule metabolic process

10. Immune system process
- Activation of immune response
- Immune effector process
- Immune response
- Immune system development
- Leukocyte activation
- Leukocyte migration

Protein classes

1. Cytoskeletal protein
- Actin family cytoskeletal protein
- Microtubule family cytoskeletal protein

2. Transporter
- ATP-binding cassette (ABC) transporter
- Amino acid transporter
- Cation transporter
- Ion channel

3. Transferase
- Acyltransferase
- Glycosyltransferase
- Kinase
- Methyltransferase
- Nucleotidyltransferase
- Phosphorylase
- Transaminase

4. Oxidoreductase
- Dehydrogenase
- Oxidase
- Oxygenase
- Peroxidase
- Reductase

5. Lyase
- Cyclase

6. Cell adhesion molecule
- Immunoglobulin superfamily cell
- Adhesion molecule

7. Ligase
- Aminoacyl-tRNA synthetase
- Ubiquitin-protein ligase

8. Nucleic acid binding
- DNA binding protein
- RNA binding protein
- Nuclease

9. Signaling molecule
- Cytokine
- Growth factor
- Membrane-bound signaling molecule

10. Enzyme modulator
- G-protein modulator
- G-protein
- Kinase modulator
- Protease inhibitor

11. Calcium-binding protein
- Annexin
- Intracellular calcium-sensing protein
-

12. Defense/immunity protein
- Antibacterial response protein
- Immunoglobulin receptor superfamily

13. Hydrolase
- Deacetylase
- Deaminase
- Esterase
- Glycosidase
- Lipase
- Phosphatase
- Protease
- Pyrophosphatase

14. Transfer/carrier protein
- Apolipoprotein

15. Membrane traffic protein
- Snare protein
- Membrane trafficking regulatory protein

16. Transcription factor
- Basic helix-loop-helix transcription factor
- Basic leucine zipper transcription factor
- Helix-turn-helix transcription factor
- Immunoglobulin fold transcription factor
- Transcription cofactor
- Zinc finger transcription factor

17. Cell junction protein
- Tight junction

18. Structural protein
- Myelin protein

19. Receptor
- G-protein coupled receptor
- Cytokine receptor
- Protein kinase receptor

20. Extracellular matrix protein

21. Transmembrane receptor regulatory/adaptor protein

22. Surfactant

23. Storage protein

24. Isomerase

25. Viral protein
