# Supplementary material for: Identification and Classification of Differentially Expressed Genes and Network Meta-Analysis Reveals Potential Molecular Signatures Associated With Tuberculosis
Source: Front Genet. 2019 Nov 4;10:932. doi: 10.3389/fgene.2019.00932 (PMC6844239; doi:10.3389/fgene.2019.00932)
Supplement: Supplementary Data 3 — (A) Community Finding (Network cluster Analysis) script in R. (B) LCP-correlation and LCP-decomposition-plot (LCP-DP approach to estimate the network compactness) method and MATLAB commonds. [file DataSheet_3.pdf]

(A). Community Finding (Network cluster Analysis)

```
setwd("G:/aftab")
library("igraph")
library("MASS")
file <- read.delim("main_ntwrk.sif",header=FALSE)
mat <- as.matrix(file)
dim(mat)
mat <- mat[,-2]
dim(mat)
graph<-graph_from_edgelist(mat, directed = FALSE)
adj = as_adj(graph)
write.matrix(adj, file="adj_sc.txt")
motifs(graph,3)
lec <- cluster_leading_eigen(graph)
sizes(lec)
lec
Subntwrk_1 <- lec[1]
Subntwrk_2  <- lec[2]
Subntwrk_3  <- lec[3]
Subntwrk_4  <- lec[4]
.

.
Subntwrk_n   <-lec[n]
dput (Subntwrk_1, file=" Subntwrk_1.txt")
dput (Subntwrk_1, file=" Subntwrk_1.txt")
dput (Subntwrk_1, file=" Subntwrk_1.txt")
dput (Subntwrk_1, file=" Subntwrk_1.txt")
.
.
.
.
dput (Subntwrk_n, file=" Subntwrk_n.txt")
community(lec)
plot(graph)
```

R- script

(B). LCP-correlation and LCP-decomposition-plot (LCP-DP approach to estimate the network compactness)

```
Function [LCPcorr, CN,LCL] = LCPcorr_and_LCDP(x,par)
% Evaluation of LCP-correlation (LCPcorr) and LCP-decomposition-plot(LCPDP) for a given network
% x: adjacency matrix of the network
% par: indicates the use of parallel computing. It is recommended only for networks with millions of nodes or very dense network topologies.
% par accepts the following values only: 0 (serial computing) or 1 (parallel computing)
% LCPcorr: LCP-correlation
% CN: list of common neighbours for each edge in the network
% LCL: list of local-community-links for each edge in the network
% Result_LCP_evaluation.mat: this MATLAB file containing the output variables is saved in the current folder
% LCPcorr_and_LCDP(x) or LCPcorr_and_LCDP(x,0): computing without parallelization
% LCPcorr_and_LCDP(x,1): computing with parallelization

*****************************************************************************************************************************

if nargin==1, par=0; end
x=full(max(x,x'));
x(eye(size(x))==1)=0;
x=logical(x);
[e,r]=find(triu(x==1,1));
w=[e r]; clear e r
```

MATLAB- Script

```
s=size(w,1);
jj=0; kb=0.3;
cn=zeros(s,1);
lcl=cn;
ne=cell(size(x,1),1);
if par==0
[cn,lcl]=computa(w,x,ne,cn,lcl,jj,s,kb);
elseif par==1
[cn,lcl]=computa_parallel(w,x,ne,cn,lcl,s);
else
disp('%')
disp('Error: Invalid value for "par" argument')
disp('"par" accepts the following values only: 0 (serial computing) or 1 (parallel computing)')
disp('%')
return
end
clear x ne w
figure
plot(cn,sqrt(lcl),'.r')
CN=cn; LCL=lcl; % output
lcl = lcl(cn>0);
cn = cn(cn>0);
if isempty(cn) || sum(lcl)==0 || numel(unique(cn))==1 || numel(unique(lcl))==1
    LCPcorr=0;
else
LCPcorr = corr(cn,lcl); % output
end
title(['LCP-correlation = ',num2str(LCPcorr)])
xlabel('CN'), ylabel('sqrt(LCL)')
save('result_LCP_evaluation','LCPcorr','CN','LCL')
```

• Prediction of network-links using neighbourhood-based indices

```
function[cn,pa,aa,ra,jc,car,cpa,caa,cra,cjc,lcl,R,fsw,cdd,cddp,ig1]= neighbourhood_link_predictors(x)
% INPUT
% x: adjacency matrix of the network
% OUTPUT
% cn => [node1, node2, ranking]; the ranking of all candidate links,
% which are missing links (or non-adjacent nodes) in the original network;
% the same kind of output applies for the other indices pa,aa,ra,jc,car,cpa,caa,cra,cjc,lcl,R,fsw,cdd,cddp,ig1
% EXAMPLE
%[cn,pa,aa,ra,jc,car,cpa,caa,cra,lcl,R,fsw,cdd,cddp,ig1]=neighbourhood_link_predictors(x)

% EXECUTION TIME EXAMPLE
% Network with 4385 nodes and 12234 interactions; computer with Intel 4 cores 2.13GHz processor,
% 8GB RAM: time execution around 60 minutes

x=full(max(x,x'));
x(eye(size(x))==1)=0;
x=logical(x);
[cn,pa,aa,ra,jc,car,cpa,caa,cra,cjc,lcl,R,fsw,cdd,cddp,ig1]=prediction(x);
```
