# Supplementary material for: Identification and Classification of Differentially Expressed Genes and Network Meta-Analysis Reveals Potential Molecular Signatures Associated With Tuberculosis
Source: Front Genet. 2019 Nov 4;10:932. doi: 10.3389/fgene.2019.00932 (PMC6844239; doi:10.3389/fgene.2019.00932)

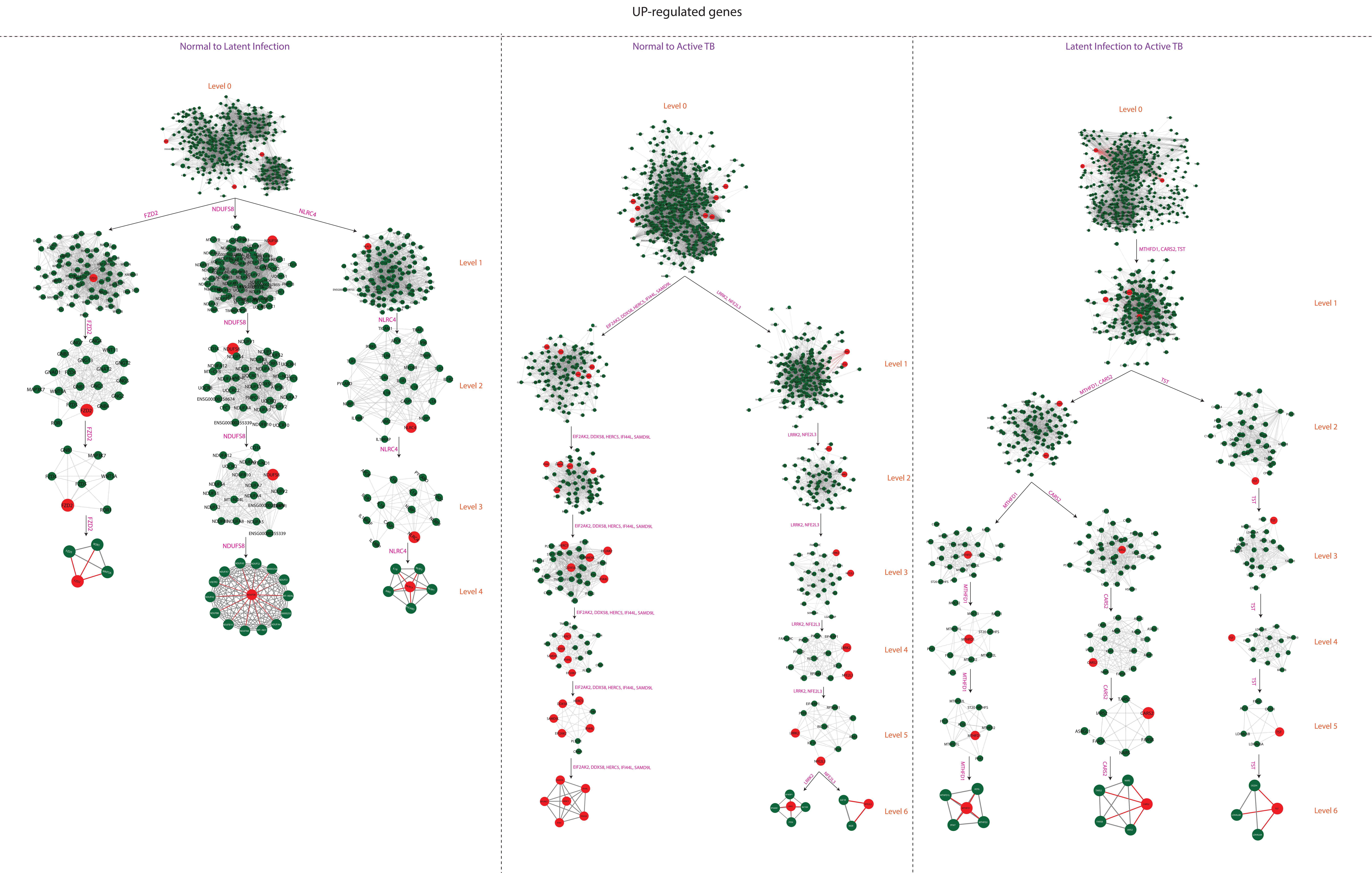
UP-regulated genes
Normal to Latent Infection
Normal to Active TB
Latent Infection to Active TB
Level 0
Level 1
Level 2
Level 3
Level 4
Level 5
Level 6
FZD2
NDUFS8
NLRC4
EIF2AK2, DDX58, HERC5, IFI44L, SAMD9L
LRRK2, NFE2L3
MTHFD1, CARS2, TST
MTHFD1, CARS2
TST
MTHFD1
CARS2

Down- regulated genes

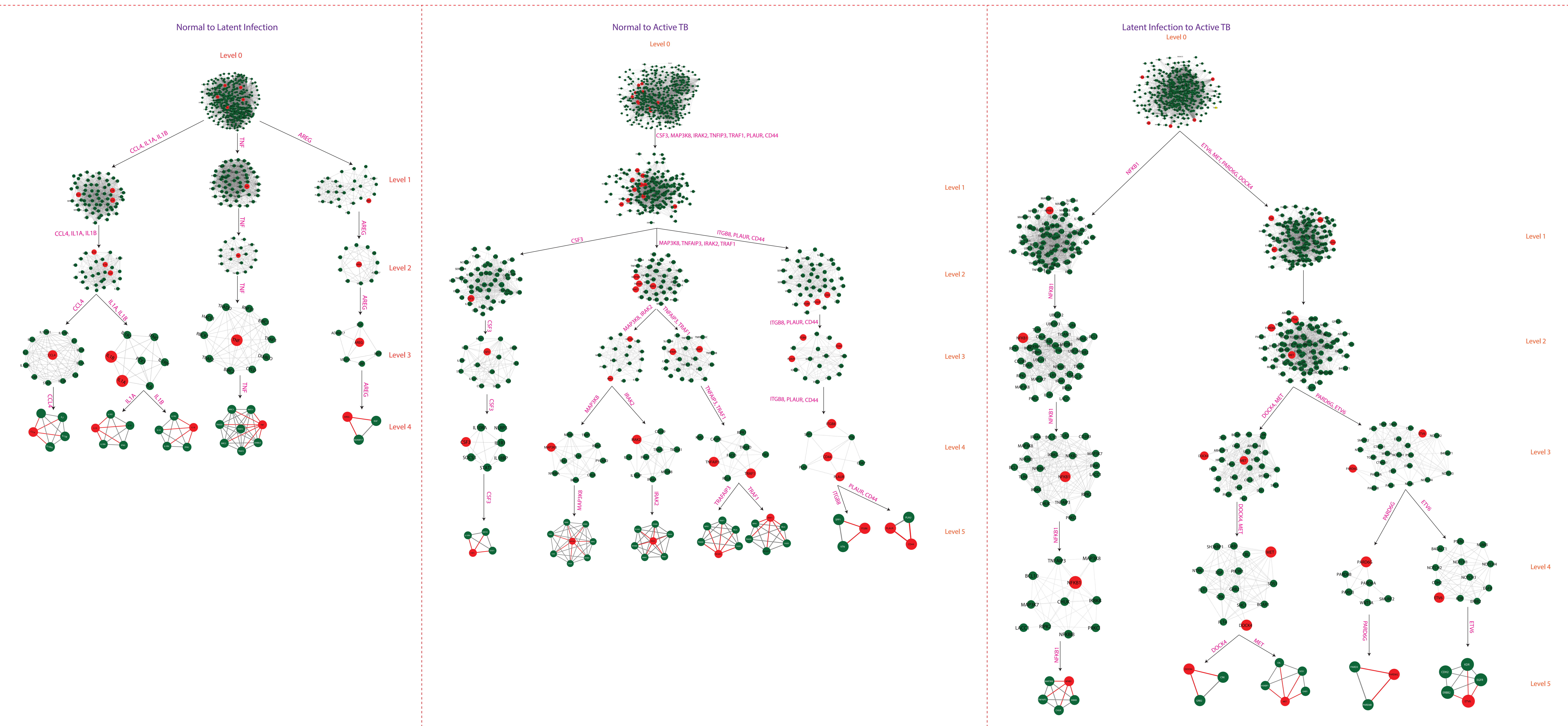

Supplement: Supplementary Data 2 — The modular path of important hub genes from complete network to motif level with the structures of modules/sub-modules at various levels. [file DataSheet_2.pdf]
